# The endosomal neuronal proteins Nsg1/NEEP21 and Nsg2/P19 are itinerant, not resident proteins of dendritic endosomes

**DOI:** 10.1038/s41598-017-07667-x

**Published:** 2017-09-05

**Authors:** Chan Choo Yap, Laura Digilio, Lloyd McMahon, Bettina Winckler

**Affiliations:** Department of Cell Biology, University of Virginia, Charlottesville, VA 22908 USA

## Abstract

Membrane traffic critically regulates most aspects of neuronal function. Neurons express many neuronal-specific proteins that regulate membrane traffic, including the poorly understood small transmembrane proteins neural-specific gene 1 and 2 (Nsg1/NEEP21 and Nsg2/P19). Nsg1 has been implicated in regulating endosomal recycling and sorting of several important neuronal receptors. Nsg2 is largely unstudied. At steady-state, Nsg1 and Nsg2 only partially co-localize with known endosomal compartments, and it was suggested that they mark a neuronal-specific endosome. Since Nsg1 localizes to and functions in the dendritic endosome, we set out to discover how Nsg1 and Nsg2 localization to endosomes is regulated in primary rat hippocampal neurons, using quadruple immunolocalization against endogenous proteins, live imaging of dendritic endosomes, and interference approaches against the endosomal regulators Rab5 and Rab7. In contrast to previous conclusions, we now show that Nsg1 and Nsg2 are not resident endosomal proteins, but traffic rapidly from the cell surface to lysosomes and have a half-life of less than two hours. Their partial co-localization with canonical endosomal markers thus reflects their rapid flux towards degradation rather than specific targeting to a singular compartment. These findings will require rethinking of how this class of endosomal proteins regulates trafficking of much longer-lived receptors.

## Introduction

The neural-specific genes Nsg1/NEEP21 and Nsg2/P19 are small transmembrane proteins highly expressed specifically in neurons^1–3^. The functions of these proteins are poorly understood, but they are found on the trans-Golgi network (TGN) and in somatodendritic endosomes^4–7^. More is known about Nsg1 than Nsg2. AMPA receptors, the cell adhesion molecule L1, and neurotensin receptors are all mislocalized and affected in their recycling^5, 8–10^ after Nsg1 knock-down. In addition, Nsg1 binds βAPP^11^. Interestingly, knockdown of Nsg1 leads to increased amyloidogenic processing. The similar trafficking effects on multiple receptors suggest that Nsg1 plays an important, but poorly understood role in dendritic endosomal sorting and transport. Nsg1 and Nsg2 are co-expressed in many (but not all) neuronal cell types in culture and in the brain^12, 13^. Since the site of action of Nsg1 is the endosome^5^, we sought to understand the pathways and regulation of its own localization to the endosome. Given the crucial relevance of disruption of endolysosomal trafficking in multiple neurodegenerative diseases, more fully understanding the cell biology of neuronal endosomes and their specific regulators has wide-reaching implications.

Previous work had identified Nsg1 as a somatodendritic endosomal membrane protein and its localization and trafficking were characterized^4^. The findings supported a type I topology for Nsg1 (N-terminus is lumenal/extracellular). Based on this conclusion, an antibody directed against the N-terminus of Nsg1 was used to investigate the exact localization and trafficking of Nsg1: Nsg1 was not detectable on the cell surface, was not endocytosed into endosomes, and was thus proposed to be trafficked to endosomes directly from the TGN^4^. The Norstrom lab recently discovered that Nsg1 mostly exists as a type II membrane protein^14^, in contrast to the previous publication^4^. We have confirmed the existence of a type II population for both Nsg1 and Nsg2 in neurons. This new finding allowed us to fully investigate the transport of Nsg1 to endosomes using C-terminal directed (extracelluar) antibodies. In addition, we extended our analysis to the mostly unstudied family member Nsg2^1^.

Our results show that both Nsg1 and Nsg2 traverse the plasma membrane and arrive in endosomes from the plasma membrane via endocytosis. New tools allowed us to follow the subsequent fate of endocytosed Nsg1 and Nsg2. The literature suggested the model that Nsg1 was a resident protein of a specialized dendritic endosome^4, 5^, but our new ability to experimentally follow the routing of endocytosed Nsg1 and Nsg2 revealed that they are not resident proteins of dendritic endosomes, but rather itinerant proteins which traverse canonical early endosomes after endocytosis and then rapidly enter degradative compartments. Their steady-state distribution in multiple non-degradative dendritic endosomes thus does not reflect any specific targeting mechanism for a resident endosomal protein, but their rapid endosomal flux towards degradation. Given the previous conclusion that Nsg1 was a stable resident protein of endosomes and was specifically targeted and retained there, these findings are surprising and will require mechanistic rethinking of how this class of endosomal proteins regulates trafficking of longer-lived receptors, such as L1 and GluA2.

## Results

### Nsg2 exists as a type II membrane protein which enters dendritic early endosomes from the cell surface

It was recently reported that Nsg1 could be detected on the plasma membrane in cultured neurons in a type II topology (N-terminus is cytoplasmic). A type I topology, which had been published previously^4^, was not apparent. In addition, type II-oriented Nsg1 entered endosomes from the cell surface^14^. In order to create the toolset necessary to decisively and unambiguously follow trafficking of endogenous Nsg1 and Nsg2 in primary neurons, we raised new antibodies against Nsg1 and Nsg2 (see Materials and Methods). A new commercial antibody of high quality against Nsg2 also became available which we recently validated and used to characterize the expression of Nsg2 in the mouse brain^13^. The validation of the goat-anti Nsg1 antibody which was not previously published is shown in Suppl. Figure S1. Only Nsg1-Em, but not Nsg2-GFP, was recognized by the antibody.

**Figure 1:**
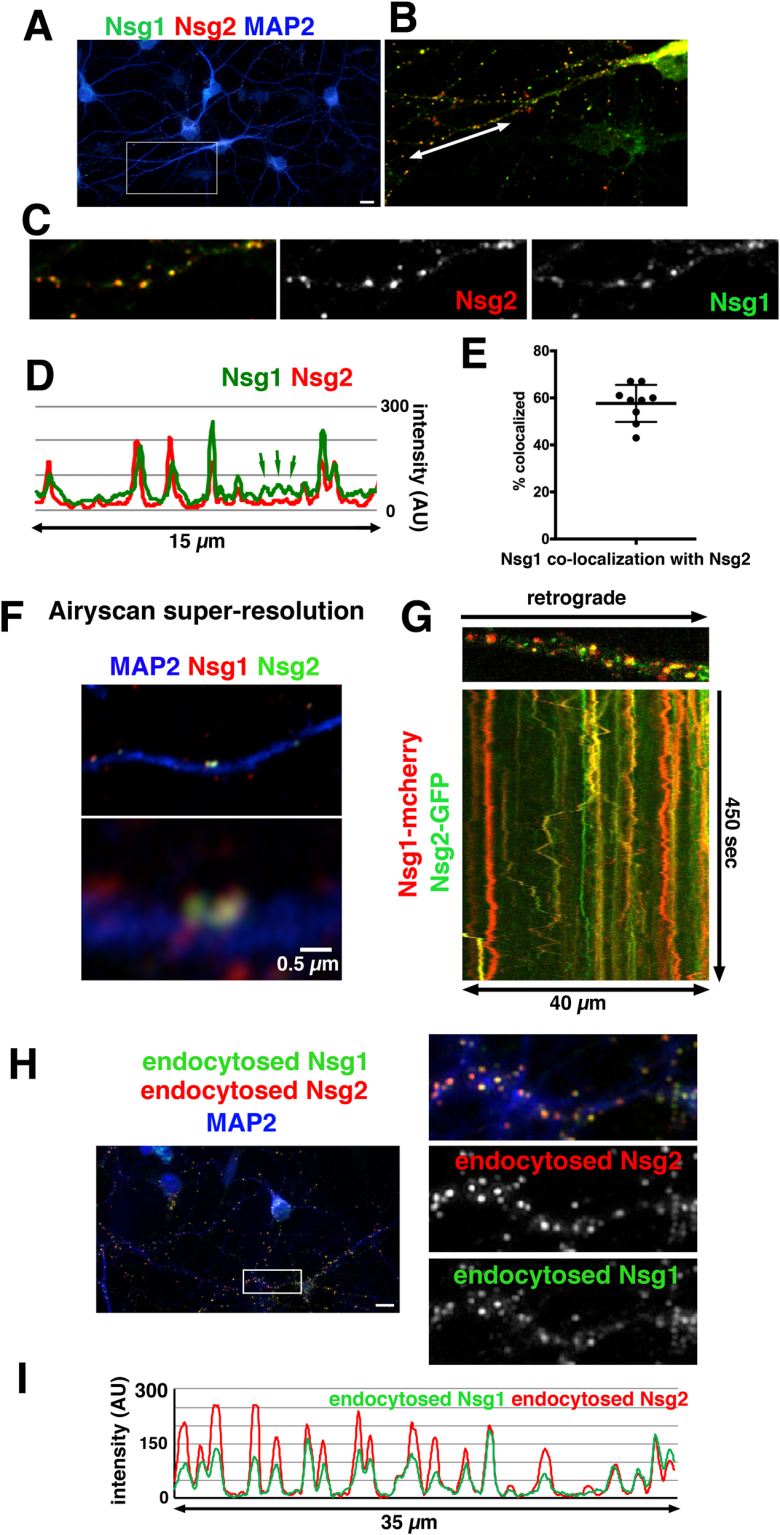
Nsg1 and Nsg2 are type II membrane proteins and endocytose from the cell surface into early endosomes. (**A–F**) Nsg1 and Nsg2 co-localize substantially. (**A**) DIV9 neurons were fixed and stained against MAP2 (blue) and endogenous Nsg1 (green) and Nsg2 (red). Bar = 10 µm. The boxed region is shown larger in (**B**). Co-localization is extensive in the zoomed-in panel (**B**). (**C**) shows a small stretch of dendrite (along arrow in B) with individual channels shown separately. A line scan is shown in (**D**). Even though co-extensive peaks are prevalent, some non-colocalizing puncta are evident (green arrows). These tend to be smaller and fainter compartments. (**E**) The extent of co-localization was quantified for endogenous Nsg1 and Nsg2 from confocal Z-stacks. Each data point corresponds to multiple dendrites in a single microscopy field containing multiple neurons. (**F**) Airyscan super-resolution imaging was used to obtain improved spatial resolution. A dendritic segment (MAP2-positive; blue) is shown with a zoomed panel below. Endosomes are crisply resolved using Airyscan. Co-incidence of Nsg1 and Nsg2 is high in endosomes which contain both proteins, but some puncta contain only one or the other (red only). (**G**) Dual live imaging of DIV9 neuron after transfection with Nsg1-mcherry and Nsg2-GFP. A still frame of one dendritic segment is shown with the kymograph displaying time on the y-axis below. Even though the relative ratios of Nsg1 and Nsg2 differ in distinct endosomes, they are greatly co-localizing to the same endosomes. (**H**) Nsg2 also exists as a type II membrane protein. Endocytosis of endogenous Nsg2 (red) and Nsg1 (green) can be detected using C-terminal directed antibodies for live uptake. The boxed region is shown enlarged on the right and a line scan along it is shown in (**I**). The line scan illustrates near-complete coincidence of peaks (**I**). Bar = 10 µm.

We first determined where Nsg2 localized in comparison to Nsg1, whose localization was previously described^4–6, 15^ (Fig. 1A,B). Co-staining revealed extensive co-localization of Nsg1 and Nsg2 both in the soma and also in numerous puncta along dendrites (Fig. 1C). Co-staining with TGN38 confirmed that Nsg2 in the soma localized to the TGN, similarly to what has been described for Nsg1 (Suppl. Figure S2). The punctate dendritic staining largely corresponded to various endosomal compartments (see detailed characterization below). In order to assess co-localization, we made line scans along dendrites (as in refs 16–18) as well as quantified co-localization (Fig. 1D,E). Co-localization was easily observed in coincident peaks on the line scan and appeared to be about 60%. The overlapping compartments tended to be the larger and brighter ones whereas the fainter compartments often contained only one of the two proteins (see small green arrows in Fig. 1D). Additionally, we imaged neurons stained against MAP2, endogenous Nsg1 and Nsg2 with super-resolution Airyscan microscopy. We found close overlap of the two proteins in many endosomes at this higher resolution as well (Fig. 1F). Lastly, we carried out dual live imaging of Nsg1-mcherry and Nsg2-GFP in dendrites and saw extensive co-localization in both stationary and motile endosomes (Fig. 1G).

Given the confusion resulting from the contrasting conclusions for Nsg1 topology, we asked whether both Nsg1 and Nsg2 were detectable as type II membrane proteins on the surface, in our hands, and able to endocytose from there. Steady state surface staining using Ct-directed antibodies against endogenous Nsg1 and Nsg2 were exceedingly faint, but could be weakly detected (Suppl. Figure S3). We thus carried out a simultaneous endocytosis assay using C-terminal antibodies against endogenous Nsg1 and Nsg2, fixed and counterstained against MAP2. We detected extensive Nsg2 puncta that were overwhelmingly co-localized with endocytosed Nsg1 (Fig. 1H), demonstrating that the C-terminus of Nsg2 was accessible on the cell surface and that Nsg2 endocytosed into somatodendritic endosomes, similarly to Nsg1. A line scan is shown as well for easier comparison of co-localizing peaks (Fig. 1I). The transient appearance of Nsg1 and Nsg2 on the surface prior to endocytosis could thereby be demonstrated using endocytosis assays. We thus found that both endogenous (Fig. 1H) and C-terminally tagged versions (data not shown) of both proteins existed as type II membrane proteins in neurons and that they trafficked to endosomes via endocytosis from the cell surface.

### Rab5 is associated with endocytosed pools of Nsg1 and Nsg2

**Figure 2:**
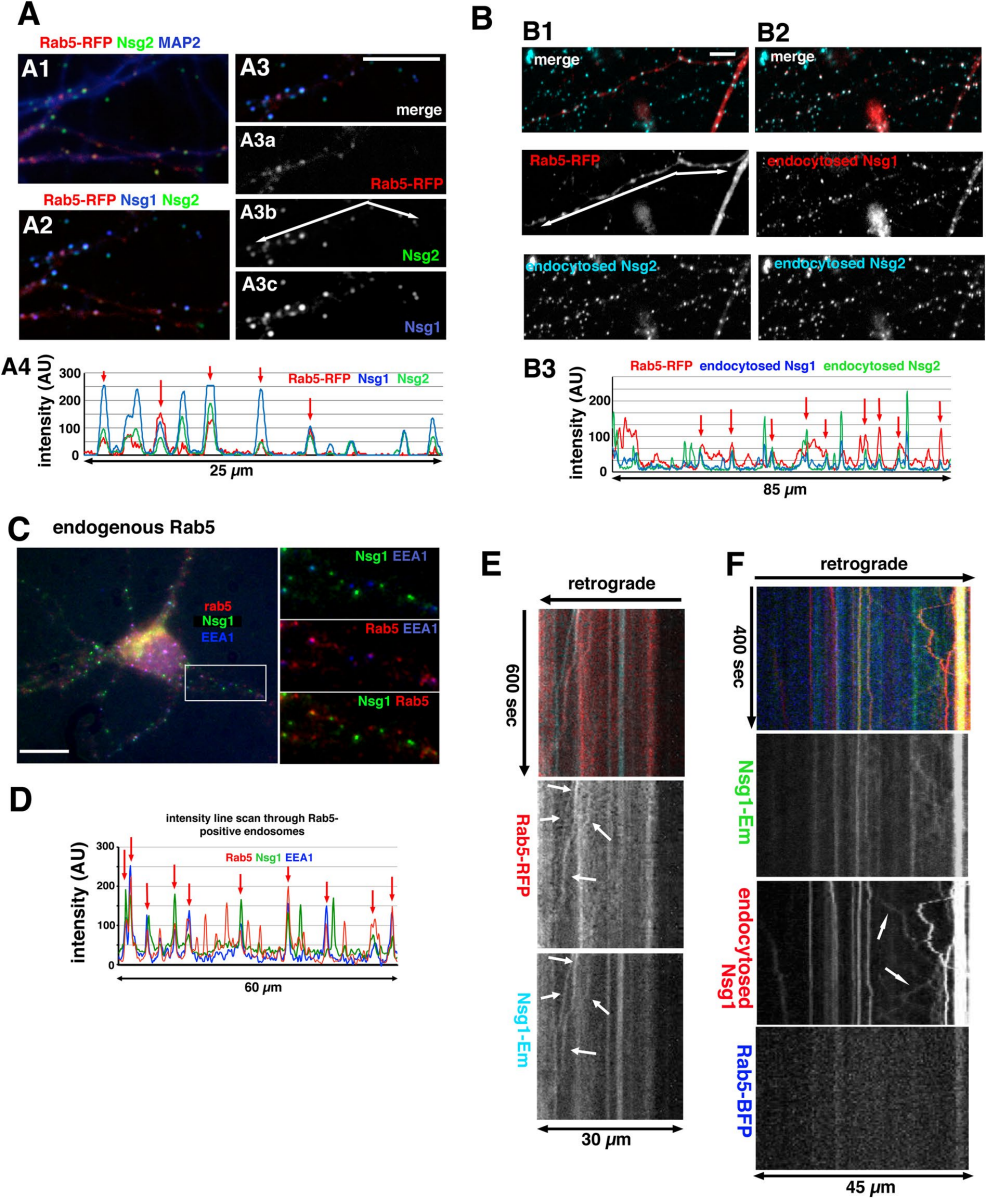
Nsg1 and Nsg2 endocytose into Rab5-positive early endosomes. (**A**–**D**) Nsg1 and Nsg2 are found in Rab5-positive early endosomes. (**A**,**B**) Endogenous Nsg1 and Nsg2, either at steady-state (**A**) or after endocytosis from the cell surface (**B**) co-localize with RFP-Rab5. (**A**) Quadruple staining against MAP2 (A1), Nsg1, Nsg2, RFP-Rab5 (A2) was carried out to visualize endosomal populations in dendrites. A close-up of one dendrite is shown in (A3) with each channel shown singly in individual panels for easier comparison. Bar = 10 µm. A line scan along the arrow shown in (A3b) is shown in (A4). Red arrows point at RFP-Rab5 positive early endosomes. (**B**) Simultaneous endocytosis assays for Nsg1 and Nsg2 were carried out in neurons transfected with RFP-Rab5 and cells counterstained for MAP2 to identify dendrites. The MAP2 channel is not shown. The RFP-Rab5, endocytosed Nsg1 and endocytosed Nsg2 channels are shown combinatorially in B1 and B2. Endocytosed Nsg2 co-localizes with RFP-Rab5 (B1). Bar = 10 µm. Endocytosed Nsg1 and Nsg2 are endocytosed into the same endosomes (B2). A line scan along the RFP-Rab5 positive dendrite (arrow in B1) is shown in (B3) for the three channels. Red arrows point at RFP-Rab5 positive early endosomes. (**C**,**D**) Staining against endogenous Rab5, EEA1 and Nsg1 show co-localization of Nsg1 in Rab5-positive endosomes (**C**). Bar = 10 µm. The boxed area is shown in dual channel combinations in the right panels. Line scan (**D**) along one dendrite is shown for easier comparison of peaks. Red arrows point at Rab5-positive early endosomes. (**E**,**F**) Live imaging of Nsg1 with Rab5 in dendrites shows partial co-localization. Kymographs are shown with the retrograde direction indicated by the arrow above. Time and distance are as marked. (**E**) DIV8 neurons were transfected with Nsg1-Em (aqua) and RFP-Rab5 (red) and live imaging carried out 48 hours later. Partial co-localization was observed, including occasional co-motility of Nsg1-Em and RFP-Rab5 puncta (arrows). We note that co-motility was infrequent. (**F**) Live imaging of Nsg1-Em (green), BFP-Rab5 (blue) and endocytosed Nsg1 (Alexa594-anti-Em antibody; red). Partial co-localization of endocytosed Nsg1 with BFP-Rab5 was observed. Bi-directional motility of endocytosed Nsg1 was observed (arrows), but usually BFP-Rab5 was not present on the motile compartment.

Rab5 resides in early endosomes and regulates endosomal trafficking into the early endosome^19, 20^. Since both Nsg1 and Nsg2 were found to endocytose (Fig. 1H), we next tested if Rab5 regulated localization and trafficking of Nsg1 and Nsg2. We found that both steady-state (Fig. 2A) and endocytosed pools (Fig. 2B) of both Nsg1 and Nsg2 showed distinct but partial overlap with RFP-Rab5. Red arrows point at coinciding peaks with Rab5-RFP in the line scans shown in Figs 2 A 4 and 2B3. We then stained against endogenous Rab5 and Nsg1 together with EEA1 to validate the localization observed with overexpressed RFP-Rab5 (Fig. 2C). We found that there was precise co-localization of early endosomes containing both EEA1 and Rab5 with a subset of Nsg1 (Fig. 2D; red arrows). The co-incident peaks can be readily appreciated on the intensity line scan shown in Fig. 2D. Expression of WT RFP-Rab5 thus did not change the staining patterns of Nsg1 or Nsg2.

Secondly, we carried out live imaging of Nsg1-Em and RFP-Rab5 and observed partial co-localization in stationary endosomes and occasionally in motile endosomes (arrows, Fig. 2E). In order to investigate whether the co-localizing Nsg1 populations were derived from the endocytosing surface pool of Nsg1, we expressed Nsg1-Em with BFP-Rab5 and then carried out a live endocytosis assay using an Alexa594-coupled antibody against the extracellular Emerald tag on Nsg1-Em. Triple live imaging showed a substantial number of motile endosomes containing endocytosed Nsg1-Em (Fig. 2F; red). Analysis of the direction of motility revealed that endocytosed Nsg1 moved bi-directionally in dendrites (see arrows in Fig. 2F). We counted endosomes containing endocytosed Nsg1 from 7 cells (98 total endosomes) and found 267 motile events (movements of >2 µm). Many Nsg1-containing endosomes thus reversed direction at least once during the course of the imaging. Of all the moving events scored, 112 were anterograde and 155 were retrograde. Endosomes containing endocytosed Nsg1 thus moved bi-directionally in dendrites with only a slight bias towards the retrograde direction. We also note that only very occasionally did the motile endocytosed Nsg1 compartment contain BFP-Rab5. Nsg1 and Nsg2 thus entered Rab5-positive endosomes after endocytosis, but mostly moved in a Rab5-negative endosome.

### Rab5 regulates endocytosis of Nsg1 and Nsg2

**Figure 3:**
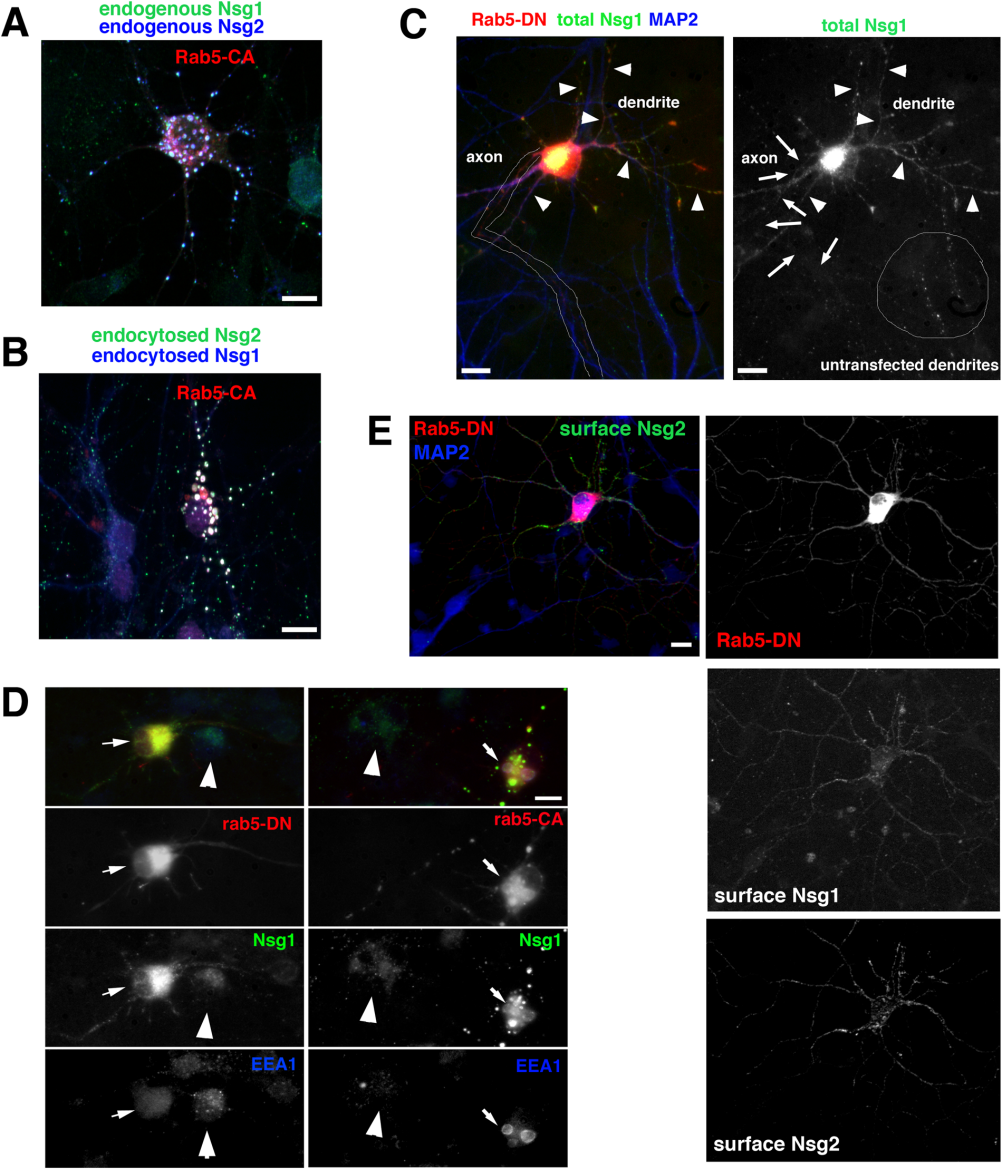
Rab5 regulates Nsg1 and Nsg2 trafficking. (**A**,**B**) Expression of Rab5-CA (constitutively active) (red) causes coalescence of endogenous steady-state Nsg1 (green) and Nsg2 (blue) (**A**) and of endocytosed Nsg1 (blue) and Nsg2 (green) (**B**) into enlarged endosomes containing Rab5-CA. Bar = 10 µm. (**C**) Neuron expressing Rab5-DN (red) shows diffuse staining of endogenous Nsg1 (green) reminiscent of surface localization. Dendrites from a non-transfected neuron (MAP2-positive processes (blue) on bottom) show punctate endosomal staining typical for Nsg1 (encircled). The Nsg1 channel is shown separately on the right. Axons are outlined (left panel) or marked by arrows (right panels), and dendrites of the transfected cell are marked by arrowheads. Bar = 10 µm. Equivalent experiments for both total endogenous Nsg1 and Nsg2 are shown in Suppl. Figure S4. (**D**) Neurons expressing Rab5-DN or Rab5-CA (red) were counterstained with Nsg1 (green) and the early endosome protein EEA1 (blue). In each panel, the transfected cell is marked with an arrow. Non-transfected cells are visible for comparison in the same field (arrowhead). Additional proteins present in endosomes which accumulate in enlarged endosomes induced by Rab5-CA are shown in Suppl. Figure S5. Only Nsg1 and EEA1 change distribution when Rab5-DN (left hand panels) is expressed. Bar = 10 µm. (**E**) Expression of Rab5-DN (red) causes accumulation of Nsg1 and Nsg2 on the cell surface. Neurons expressing Rab5-DN (red) were surface-stained live before fixation with antibodies directed against the Ct of endogenous Nsg1 and Nsg2. MAP2 counterstain (blue) is shown in the top panel together with Rab5-DN (red) and surface Nsg2 (green). Surface staining of Nsg1 (middle panel) and Nsg2 (bottom panel) is observed in neurons expressing Rab5-DN (arrow), but little surface expression is detectable in non-transfected cells present in the same field. Single channel images are shown. Bar = 10 µm.

In order to test if Rab5 regulated the trafficking of Nsg1 and Nsg2, we expressed either a constitutively active Rab5 (Rab5-CA: Rab5-Q79L) or a dominant negative Rab5 (Rab5-DN: Rab5-S34N). Rab5-CA leads to excessive fusion of endosomes and accumulation of many receptors and regulators of endosomes, including Nsg1^4, 6^. As expected, both steady-state (Fig. 3A) and endocytosed pools (Fig. 3B) of both Nsg1 and Nsg2 accumulated similarly in enlarged somatic and dendritic endosomes in all transfected neurons, suggesting that they are trapped in Rab5-CA-containing endosomes after endocytosis from the surface. Our observations so far suggest that a large proportion of Nsg1 and Nsg2 localizes to endosomes via endocytosis from the cell surface.

We then determined the effect of expressing Rab5-DN on Nsg1 localization (Fig. 3C). The localization of Nsg1 changed strikingly in ~60% of the transfected neurons and appeared more diffuse or in very small puncta. Comparison of the transfected cell expressing Rab5-DN on top of the field with the dendrites from an untransfected cell extending into the field from the bottom (encircled) highlights the difference between the mostly punctate endosomal staining typical for Nsg1 in the untransfected dendrites and the more diffuse/small puncta staining in the transfected cell. We posit that the other 40% of transfected neurons expressed insufficient Rab5-DN to elicit a robust phenotype. Other work showed mislocalization of many dendritic receptors to axons when Rab5 function was impaired^21^. We did not observe such mislocalization in the time frame of our experiment (Fig. 3C). The diffuse Nsg1 staining in the Rab5-DN expressing neuron was restricted to soma and dendrites (MAP2-positive; arrowheads) and could not be observed in the axon (MAP2-negative; outline or arrows). The staining of total Nsg2 was similarly affected by Rab5-DN expression and became less obviously punctate (Suppl. Figure S4).

We next tested what other endogenous proteins changed localization when either Rab5-CA or Rab5-DN were expressed. We examined the distribution of the cis-Golgi protein GM130 (Suppl. Figure S5A), the TGN/endosome-cycling mannose-6-phosphate receptor (M6PR) (Suppl. Figure S5B), the early endosomal protein EEA1 (Fig. 3D), and the lysosome-associated membrane protein LAMP1 (Suppl. Figure S5C). For each set of panels, a transfected neuron and a non-transfected neuron are visible. We found that Rab5-CA led to accumulation of all proteins tested in the Rab5-CA induced enlarged endosomes, except for the Golgi-resident GM130 (right hand panels in Suppl. Figure S5). Rab5-DN, in contrast, changed only the localization of Nsg1 and EEA1 (Fig. 3D; left hand panels). Similarly to Nsg1, EEA1 was no longer enriched on punctate early endosomes in ~60% of transfected neurons, but became diffuse. This observation was consistent with previous findings that downregulation of Rab5 led to disappearance of endosomes over time^19^. Both M6PR (found in the TGN and late endosomes) and LAMP1 (found in late endosomes and lysosomes) remained associated with punctate organelles in neurons expressing Rab5-DN (Suppl. Figure S5; left hand panels).

In order to test if the more diffuse appearing staining observed in Rab5-DN expressing neurons was due to an accumulation of Nsg1 and Nsg2 on the surface or intracellularly, we stained non-permeabilized live neurons after transfection with Rab5-DN. Both endogenous Nsg1 and Nsg2 were now clearly detectable on the cell surface using C-terminal directed antibodies (Fig. 3E; arrow). This was in striking contrast to non-transfected cells (Fig. 3E) or cells expressing WT RFP-Rab5 (Fig. 2) where diffuse expression of Nsg1 and Nsg2 was not apparent in any of the transfected neurons. Faint surface staining of a non-transfected cell present in the same field was visible in Fig. 3E. In contrast to other dendritic receptors (including TfR and GluA2)^21^, we thus observed no axonal missorting of Nsg1 or Nsg2 when Rab5-DN is expressed, but rather Nsg1 and Nsg2 accumulated on the somatodendritic surface and appeared to be impaired in endocytosis.

### Nsg1 and Nsg2 largely bypass recycling endosomes

**Figure 4:**
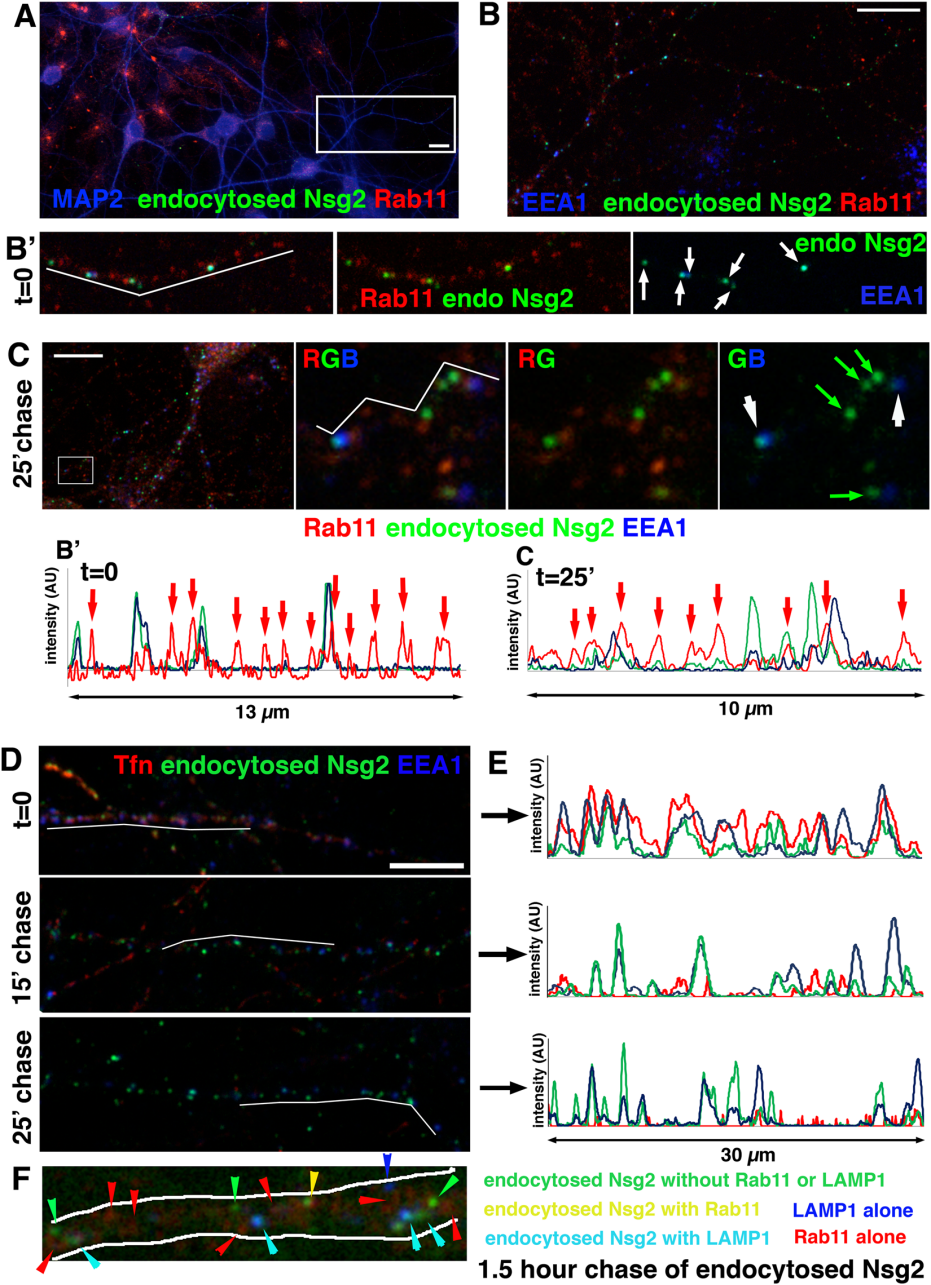
Endocytosed Nsg2 barely enters recycling endosomes. (**A–C**) An endocytosis assay with endogenous Nsg2 was carried out and cells either fixed (t = 0; **A**,**B**) or returned to the incubator for a 25 minute chase period before fixation (**C**). Bar = 10 µm. After fixation, cells were counterstained for MAP2 to mark dendrites (**A**), EEA1 to mark early endosomes, and endogenous Rab11 to mark recycling endosomes (**B**). (B’) shows one dendrite as merged images of all three channels and combinations of two channels as marked on the panels. Endocytosed Nsg2 (green) rarely co-localizes with Rab11 (red; arrowhead), but shows many instances of co-localization with EEA1 (blue; arrows). A line scan along the white line in B’ is shown. (**C**) After 25 minute chase, endocytosed Nsg2 still shows some co-localization with endosomes containing high levels of EEA1 (white arrows), but no increase in overlap with Rab11 (red) is observed (see red arrows in the corresponding line scan along the white line). Green arrows indicate endosomes containing endocytosed Nsg2 with low levels of EEA1. R = red, G = green, B = blue. Corresponding experiments for endocytosed endogenous Nsg1 are shown in Suppl. Figure S6. (**D,E**) Cy3-Tfn (red) and anti-Nsg2 antibody (green) were simultaneously fed to live cultures for 30 minutes (endocytosis assay) and then fixed at t = 0, or after 15 minute or 25 minute chase times (**D**). Bar = 10 µm. After fixation, cells were counterstained with MAP2 to identify dendrites (channel not shown) and EEA1 to identify early endosomes (blue). Since Tfn recycles rapidly, the red channel intensity for the 15 and 25 minute chase time was increased so that localization of the small number of remaining Tfn compartments could be more easily visualized in the panel. (**E**) Shows the corresponding line scans through the MAP2-positive dendrite shown in (**D**). Tfn co-localizes extensively with endocytosed Nsg2 and with EEA1 at t = 0, but then rapidly leaves the early endosomes whereas endocytosed Nsg2 remains co-localized with EEA1 throughout the chase. (**F**) Endocytosed endogenous Nsg2 was chased for 1.5 hours and then counterstained against endogenous Rab11 and LAMP1. A close-up of one representative dendrite is shown. Colored arrowheads correspond to endosomes with presence or absence of the stained markers. The color coding is indicated on the right. Turquoise arrowheads indicate endocytosed Nsg2 present in LAMP1-positive late endosomes/lysosomes.

Previous reports showed that Nsg1-containing endosomes convert from EEA1-positive early endosomes to EEA1-negative endosomes^6^. The identity of these Nsg1-positive, EEA1-negative endosomes was not determined, but could be recycling endosomes or late endosomes, or a unique uncharacterized population of endosomes. Figure 2 showed both Nsg1 and Nsg2 endocytosed into early endosomes. Our ability to follow endocytosed pools of Nsg1 and Nsg2 now for the first time allowed us to determine their subsequent fate. We thus asked if Nsg1 and Nsg2 entered recycling endosomes after endocytosis. We used two markers for the recycling pathway. The first was co-staining against endogenous Rab11 (Fig. 4A–C). The second was co-uptake of labeled transferrin which rapidly recycles through early and recycling endosomes (Fig. 4D,E).

In order to determine if Nsg2 entered recycling endosomes, we carried out an Nsg2 endocytosis assay by feeding anti-Ct Nsg2 antibody for 30 minutes. Neurons were either fixed immediately (t = 0; Fig. 4A,B) or washed and returned to the incubator for a 25 minute chase and then fixed (t = 25′ chase, Fig. 4C). Cultures were counterstained with antibodies against MAP2 to identify dendrites, EEA1 to identify early endosomes, and Rab11 to identify recycling endosomes. The MAP2-positive dendrite boxed in Fig. 4A is shown in Fig. 4B without MAP2 for comparison of the three endosomal populations. In Fig. 4B’, a zoomed-in region is shown as triple, and double channel images. Arrows point at early endosomes (EEA1-positive) containing endocytosed Nsg2. Whereas most endocytosed Nsg2 resided with EEA1 at t = 0 (Fig. 4B’), little was co-localized with Rab11. After a 25-minute chase, endocytosed Nsg2 was found in a pool partially overlapping with EEA1 (white arrows), but it did not majorly chase into Rab11-positive endosomes. Green arrows in Fig. 4C denote endocytosed Nsg2 largely devoid of EEA1 or Rab11. Line scans of Fig. 4B’ and C are shown as well.

Secondly, we carried out simultaneous endocytosis of Cy3-Tfn and anti-Ct Nsg2 antibody for 30 minutes and then fixed the neurons immediately or after a chase of 15 or 25 minutes (Fig. 4D). Early endosomes were detected with anti-EEA1 immunostaining. At t = 0, there was extensive co-localization of both endocytosed cargos (Tfn and Nsg2) with early endosomes. This can be seen by the co-extensive peaks on the line scan (Fig. 4E). After 15 minutes of chase, Tfn was already greatly reduced in intensity and number of endosomes, consistent with its rapid recycling. By 25 minutes of chase, Tfn was mostly gone from endosomes. Endocytosed Nsg2, in contrast, was still found in brightly stained endosomes which frequently co-localized with EEA1-positive early endosomes (Fig. 4D). This can be appreciated better in the line scans (Fig. 4E). We thus conclude that Nsg2 and Tfn entered early endosomes together after endocytosis, but Tfn rapidly sorted away from EEA1 and endocytosed Nsg2. We also carried out equivalent experiments using anti-Nsg1 endocytosis assays. We similarly observed low co-localization of endocytosed Nsg1 with Rab11 at t = 0 or a 25 minute chase (Suppl. Figure S6). Both endocytosed Nsg1 and Nsg2 thus remained at least partially in early endosomes for half an hour or more and overwhelmingly did not chase into Rab11-positive recycling endosomes. Co-localization of steady-state Nsg1 and Nsg2 with Rab11 was also low (see below).

**Figure 6:**
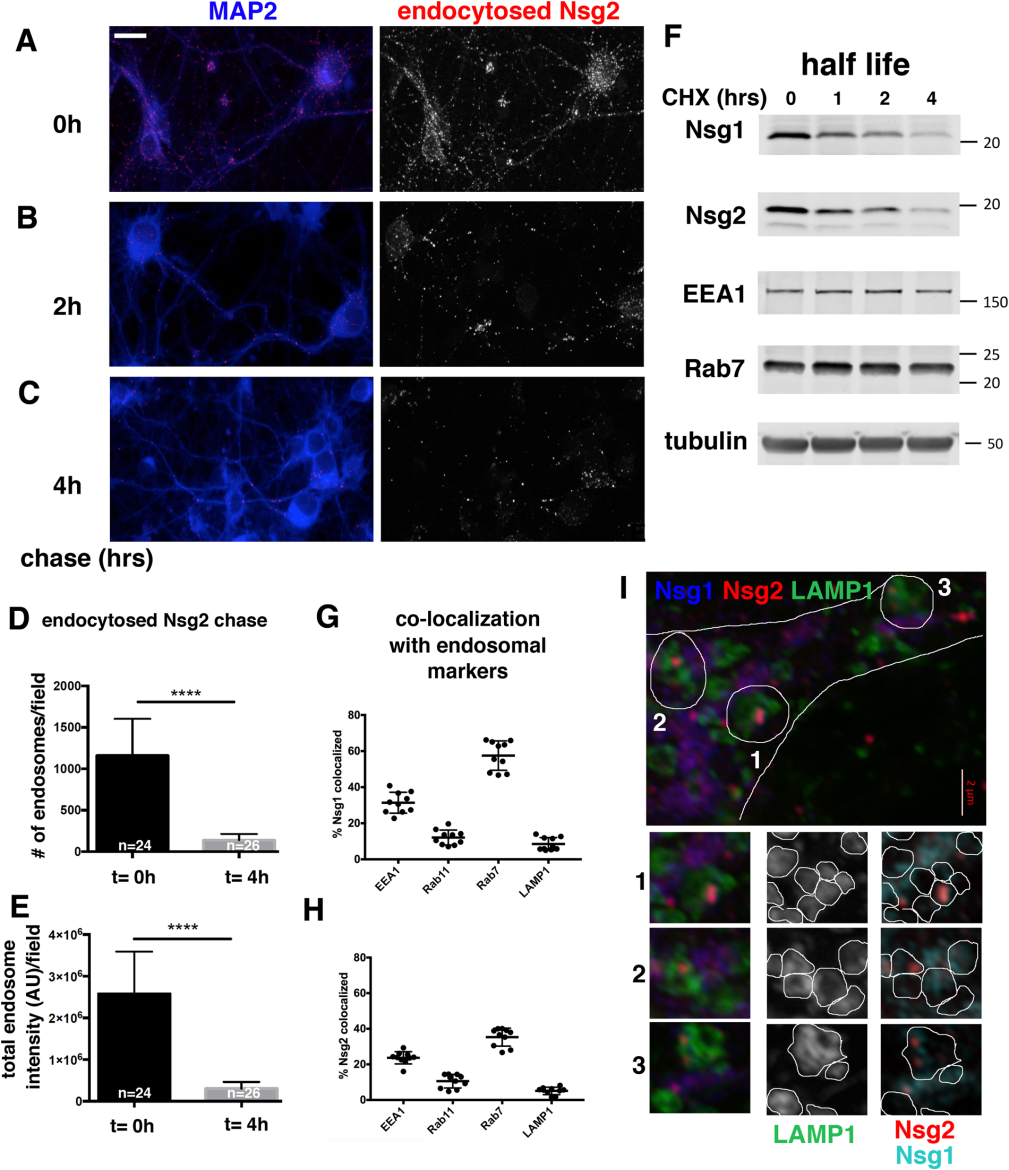
Nsg1 and Nsg2 degrade rapidly. (**A**–**C**) Neurons were fed live with anti-Nsg2 antibodies (red) to load endosomes and then washed, fixed immediately t = 0 (**A**), or chased for 2 hours (**B**) or 4 hours (**C**). MAP2 was used as a counterstain to identify dendrites (blue). The red channel alone is shown in the right panels. Very little endocytosed Nsg2 remains in cells after 4 hours of chase. (**D**,**E**) Quantification of number of Nsg2-containing endosomes (**D**) and fluorescence intensity in Nsg2-containing endosomes (**E**) at t = 0 and t = 4 hours of chase. N = number of microscopy fields quantitated in two independent experiments. The total number of cells quantified for these graphs was >600. The differences are highly significant (Mann-Whitney test; p < 0.0001). (**F**) Half-lives of Nsg1 and Nsg2 were determined by Western blotting of neuronal membrane fractions after treatment with cycloheximide (CHX) for 0, 1, 2 and 4 hours. EEA1 and Rab7 were also blotted for comparison. Both displayed much longer half-lives than Nsg1 and Nsg2. The neuronal isoform β3-tubulin is shown as a loading control to normalize across the different lysates. One representative experiment of three independent repeats is shown. Cropped blots are displayed. The full length blots are shown in Suppl. Figure S8. (**G**,**H**) Extent of co-localization of Nsg1 (**G**) and Nsg2 (**H**) with EEA1, Rab11, Rab7 and LAMP1 was quantified for ten fields of 3D reconstructed confocal stacks using Imaris software. Each data point corresponds to one microscope field containing multiple cells and up to five dendrites per field. (**I**) Co-localization of Nsg1 (blue) and Nsg2 (red) with the lysosomal marker LAMP1 (green) was visualized with 3D superresolution Airyscan microscopy. Co-localization was not striking at steady-state, but clear instances could be observed. In many examples, the interior lumen of the doughnut-shaped lysosomes could be resolved and frequently Nsg1 and Nsg2 were found inside. Three examples are circled in the panel and each is shown below as smaller insets with separate channels. Individual putative lysosomes are identifiable and were circled in the LAMP1 channel (center panels). This mask was then duplicated onto the Nsg1/Nsg2 channels (red and aqua) in the right hand panels.

### Nsg1 and Nsg2 travel in Rab7-positive late endosomes

Since endocytosed Nsg1 and Nsg2 did not majorly enter recycling endosomes after accumulation in early endosomes, we wondered if any of the endocytosed Nsg proteins would ultimately chase to late endosomes/lysosomes. We thus co-stained endocytosed Nsg2 chased for 1.5 hours with both Rab11 and LAMP1. We could detect clear examples of puncta positive for Nsg2 and LAMP1 (Fig. 4F; turquoise arrowheads). This observation suggested that endocytosed Nsg proteins might sort into Rab7-positive late endosome and ultimately degrade in lysosomes. Previous reports had concluded that Nsg1 was a resident protein of Rab4-positive, Rab5-negative early endosomes and that it was not found in degradative endosomes^4^, but Rab7 staining had not been used to look at late endosomes.

**Figure 5:**
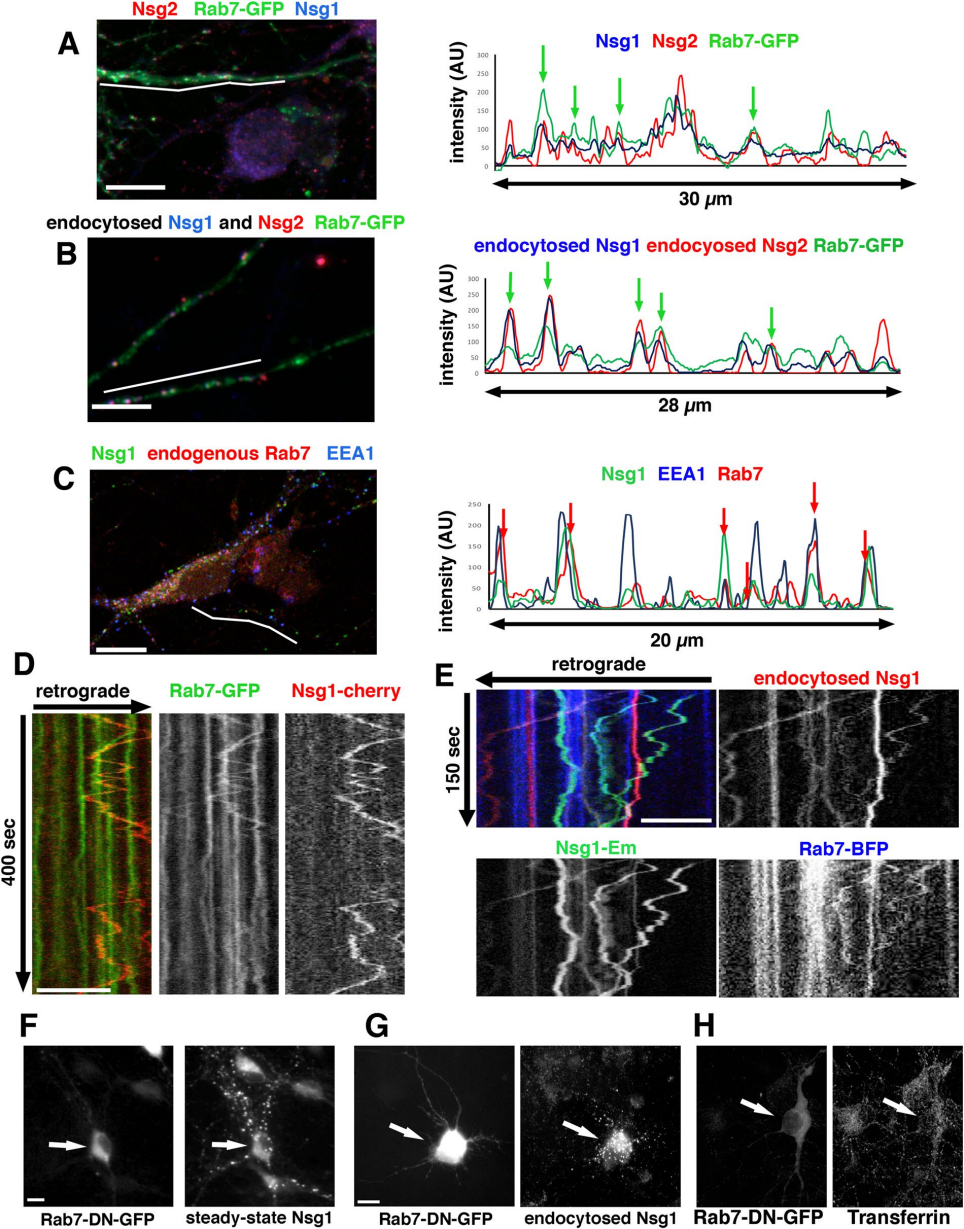
Nsg1 and Nsg2 travel in Rab7-positive late endosomes. (**A**–**C**) Co-localization of Nsg1 and Nsg2 with Rab7. MAP2 was counterstained to identify dendrites for line scans in all experiments, but the channel is not shown. The white line indicates the location of the corresponding line scans. (**A**) GFP-Rab7 (green) was transfected into DIV8 neurons and cells were counterstained against endogenous steady-state Nsg1(blue) and Nsg2 (red). Bar = 10 µm. A line scan is shown on the right. Green arrows indicate GFP-Rab7 endosomes also containing Nsg1/2. (**B**) GFP-Rab7 (green) was transfected into DIV8 neurons and simultaneous endocytosis assays were performed using anti-Nsg1 (blue) and Nsg2 (red) antibodies. Endocytosed Nsg1 and Nsg2 were detectable in Rab7-positive endosomes. Bar = 10 µm. A line scan is shown on the right. Green arrows indicate Rab7-GFP endosomes also containing endocytosed Nsg1 and Nsg2. (**C**) Confocal images of endogenous Rab7 (red), Nsg1(green) and EEA1 (blue) staining reveals partial co-localization. Bar = 10 µm. A line scan is shown on the right. Red arrows indicate Rab-7 endosomes containing Nsg1. (**D**,**E**) Live imaging of Nsg1 with Rab7 reveals co-motility. (**D**) Dual live imaging was carried out in neurons expressing GFP-Rab7 and Nsg1-mcherry. The kymograph illustrates Nsg1-mcherry in a subset of GFP-Rab7 endosomes which were often motile. A merged panel of both channels as well as each channel separately are shown. Bar = 10 µm. The position of the retrograde direction is indicated by the arrow. (**E**) Triple live imaging was carried out in neurons transfected with Nsg1-Em (total Nsg1; green) and BFP-Rab7 (blue). Endocytosis assays of Alexa568-anti-Em antibody was carried out to visualize endocytosed Nsg1 (red). The kymograph illustrates that endocytosed Nsg1 (red) co-localized with a subset of BFP-Rab7 (blue) and often moved retrogradely in Rab7-positive endosomes. Anterograde co-motility is also observed. A merged panel of all channels as well as each channel separately are shown. Bar = 10 µm. (**F–H**) Rab7-DN expression leads to accumulation of steady-state (**F**) and endocytosed Nsg1 (**G**). Nsg1 staining in the transfected neuron is brighter than in non-transfected neurons in the same field. Corresponding experiments for Rab7 knock down and Rab7-DN are shown in Suppl. Figure S7 for both endogenous Nsg1 and Nsg2. (**H**) Tfn levels were not increased in cells expressing Rab7-DN compared to non-transfected neurons. Arrows indicate transfected cell. Bar = 10 µm.

We first expressed Rab7-GFP and either co-stained against steady-state Nsg1 and Nsg2 (Fig. 5A) or carried out a simultaneous endocytosis assay with Ct-directed antibodies against both Nsg1 and Nsg2 (Fig. 5B). Line scans are shown on the right and show partial overlap with GFP-Rab7 positive endosomes (green arrows). In order to confirm this novel conclusion with a different approach we also used antibodies against endogenous Rab7. Cultured neurons were fixed and stained against MAP2 (to mark dendrites), EEA1 (to mark early endosomes), Rab7 (to mark late endosomes) and Nsg1 (Fig. 5C). A line scan through a MAP2-positive dendrite is shown on the right. Clear coinciding peaks of Rab7 and Nsg1 could be observed (red arrows). This observation suggested that a subpopulation of Nsg1 and Nsg2 resided in late endosomes.

Next, we investigated by live imaging whether Nsg1-cherry co-localized with GFP-Rab7 (Fig. 5D). We also carried out endocytosis assays against Nsg1-Em and visualized total Nsg1-Em (green), endocytosed Nsg1-Em (red) and BFP-Rab7 by triple live imaging (Fig. 5E). Nsg1-Em and endocytosed Nsg1-Em were strikingly co-localized with Rab7, including in retrogradely and anterogradely moving late endosomes.

### Interference with Rab7 function leads to accumulation of Nsg1 and Nsg2 in endosomes

Since we observed clear co-localization of Nsg1 and Nsg2 with Rab7, we tested whether interfering with Rab7 function would disturb the localization of Nsg1 and Nsg2. The dominant negative Rab7-T22N delays and reduces the degradation of LDL and EGF without interfering with endocytosis^22–24^. We thus expressed GFP-Rab7-T22N (Rab7-DN) in cultured neurons and either stained for the steady-state endogenous Nsg1 (Fig. 5F) or for endocytosed Nsg1 after anti-Nsg1 antibody feeding (Fig. 5G). We observed a striking increase in staining intensities in 70–80% of transfected neurons (arrows) for both steady-state and endocytosed Nsg1, compared to untransfected neurons. Equivalent experiments were carried out for Nsg2 and showed similar accumulation (Suppl. Figure S7). Downregulation of Rab7 by shRNA against Rab7 in neurons also resulted in accumulation of steady-state endogenous Nsg1 and Nsg2 (Suppl. Figure S7). Expression of WT Rab7-GFP did not cause substantial accumulation of Nsg1 and Nsg2 (Suppl. Figure S7). These observations suggested that Rab7-DN blocked forward trafficking of Nsg1 and Nsg2 leading to their accumulation. Consistent with reports in the literature^22^, the recycling cargo Tfn did not accumulate in neurons expressing Rab7-DN compared to non-transfected cells (Fig. 5H). Our observations suggested that Nsg1 and Nsg2 entered late endosomes after endocytosis and did not primarily recycle via recycling endosomes.

### Endocytosed Nsg1 and Nsg2 degrade rapidly

Previous work^4^ had concluded that Nsg1 was not present in lysosomes. Our data, on the other hand, showed little overlap of Nsg1 and Nsg2 with Rab11-positive recycling endosomes (Fig. 4), but substantial overlap with Rab7-positive late endosomes (Fig. 5). In addition, a subset of endocytosed Nsg2 chased into LAMP1-positive compartments (Fig. 4F), suggesting that endocytosed Nsg1 and Nsg2 might be trafficking towards a degradative fate. In addition, Rab7-DN expression led to accumulation of Nsg1 and Nsg2 (Suppl. Figure S7). We thus carried out a pulse-chase experiment after anti-Nsg2 antibody feeding (Fig. 6A–C). Neurons were subsequently washed (Fig. 6A, t = 0 h) and returned to the incubator for chase times of 2 hours (Fig. 6B) and 4 hours (Fig. 6C). By 4 hours of chase, very little endocytosed Nsg2 was detectable in neurons (Fig. 6D,E). To note, we did not observe any endocytosed Nsg2 in Golgi-like structures during the chase, indicating no retrograde trafficking from endosome back to TGN after internalization.

In order to test if the loss of signal was thus due to degradation, we determined the half lives of Nsg1 and Nsg2. Neuronal cultures were treated with cycloheximide (CHX) for 1–4 hours to inhibit new protein synthesis, and then membrane fractions were analyzed by Western blots (Fig. 6F). Both Nsg1 and Nsg2 displayed half lives of <two hours. Quantification of the Western blot band intensities showed that Nsg1 levels dropped to 47% at two hours and 28% at four hours. Nsg2 levels dropped to 40% at one hour, 26% at two hours, and 11% at four hours. These half lives are much shorter than the membrane-associated endosomal protein EEA1 (77% at two hours, 60% at four hours) (Fig. 6F). The levels of Rab7 and β3-tubulin did not decrease noticeably in four hours. In agreement with the Western blots, we also observed the staining of endogenous Nsg1 decrease in neurons treated with CHX (data not shown).

Since degradation of membrane proteins largely takes place in lysosomes, we revisited the question of whether Nsg1 and Nsg2 could be at least partially detected in lysosomes (marked by LAMP1) at steady state. We quantified co-localization of Nsg1 and Nsg2 with markers for early endosomes (EEA1), recycling endosomes (Rab11), late endosomes (Rab7), and late endosomes/lysosomes (LAMP1) from confocal stacks using Imaris software (Fig. 6 G,H). Both Nsg1 and Nsg2 showed the highest co-localization with Rab7, with very low co-localization found with Rab11 and LAMP1. These data confirmed our observations from line scans that Nsg1 and Nsg2 were found predominantly along the degradative (Rab7-LAMP1) arm of the endosomal pathway and were less prevalently found in the recycling (Rab11) arm.

Lastly, we carried out triple super-resolution Airyscan imaging against endogenous LAMP1, Nsg1, and Nsg2 (Fig. 6I). In agreement with Steiner^4^, the LAMP1 and steady-state Nsg1 staining patterns were largely distinct (Fig. 6I). In fact, the brightest LAMP1-staining in the soma was largely non-overlapping with the brightest Nsg1 and Nsg2 staining. We did nevertheless clearly detect some amount of co-localization (examples encircled in Fig. 6I). Using Airyscan microscopy, we were able to resolve the limiting and lumenal aspects of the larger somatic lysosomes which have a doughnut appearance with a clear center (insets in Fig. 6I). Interestingly, Nsg1 and Nsg2 could frequently be observed within the lumen of these lysosome. This localization suggested that Nsg1 and Nsg2 are not resident proteins of the limiting lysosomal membrane but found in the lumen, consistent with their rapid degradation.

## Discussion

Neurons are highly specialized cells with unique morphology, very large size and virtually unparalleled cellular longevity. These unique features of neurons have prompted much research into the molecular and cellular adaptations that allow for the elaboration of their highly polarized and gigantic morphology^25–28^. Neurons express many proteins in common with non-polarized cells, but there is also a large number of proteins which are particularly highly expressed in neurons. Among them are a poorly understood family of small transmembrane proteins, which was identified as being neuron-specific in their expression and was thus named “Nsg” for “neural-specific genes”^2, 3, 29^. The Nsg family of genes evolved late and is a vertebrate invention, not found in C. elegans or Drosophila^1^. They are thus likely not house-keeping proteins, but important for specialized tasks which are currently poorly elucidated. Unraveling the function of such specialized proteins is challenging, but could lead to the identification of a set of proteins that might be important in vertebrates, especially for highly evolved brain functions^29^. Knockdown approaches in cultured neurons revealed that Nsg1 (NEEP21) is involved in regulating the endosomal transport of a variety of important neuronal receptors^5, 8, 9, 11^. Understanding where Nsg1 and Nsg2 reside when carrying out these critical regulatory functions and how their localization is controlled is thus an important step towards uncovering the molecular roles of neuronal endosomal regulators.

Previous work concluded that Nsg1 was stably localized in a poorly characterized neuronal early endosome (Rab5-negative, Rab4-positive, EEA1-negative) that might be specific to neurons^4^. In contrast, we find that both Nsg1 and Nsg2 rapidly traverse both canonical early and late endosomes after endocytosis from the surface and are rapidly degraded with a half-life of less than two hours. Nsg1 and Nsg2 are thus not resident endosomal proteins. Rather, their partial co-localization with canonical endosomal markers reflects their rapid flux towards degradation. Given the previous belief that Nsg1 and Nsg2 were stable resident proteins of endosomes and were specifically targeted and retained there, these findings require substantial rethinking of how this class of endosomal proteins regulates trafficking of longer-lived receptors, such as L1 and GluA2. Our findings thus shed light on multiple fields, including endosomal regulators in neurons, the machinery for regulating the trafficking of neurotransmitter receptors in synapses or of βAPP, and the regulation of degradative flux in neurons.

### New tools uncovered trafficking dynamics of Nsg1/2 from the surface to lysosomes

Previous work suggested that Nsg1 trafficked directly to somatodendritic endosomes from the TGN and did not traverse the cell surface^4^. It was also shown that Nsg1 resided in early endosomes, but was largely absent from lysosomes. Subsequent work confirmed that about 25% of Nsg1 was found in early endosomes^5, 6, 15^, but there was a substantial pool that did not coincide with early endosomes or other known compartments. It was thus proposed that Nsg1 might be present in an endosomal compartment unique to neurons. In contrast to some of the previous work, we now report that the small transmembrane proteins Nsg1 (NEEP21) and Nsg2 (P19) reach their steady-state endosomal localization in dendrites by a Rab5-regulated endocytic event from the cell surface. We further show that Nsg1 and Nsg2 are not stable resident endosomal proteins, but rapidly travel to lysosomes for degradation after traversing early and late endosomes. They are thus itinerant endosomal proteins. These observations change previous conclusions about how the steady-state endosomal localization of these neuronal-specific proteins is regulated. Our new conclusions were made possible by the recent discovery that Nsg1 is a type II membrane proteins^14^. Our work is also the first to look at Nsg2 trafficking. We find Nsg2 to also be type II and to co-localize and behave quite similarly to Nsg1. Interestingly, we recently showed that Nsg1 and Nsg2 are not identically expressed in the brain and show some unique expression patterns^13^. Further work is needed to unravel to what extent Nsg1 and Nsg2 have redundant or unique functions.

### Interference with Rab5 leads to accumulation of Nsg1 and Nsg2 on the surface

Our data show that Nsg1 and Nsg2 were found transiently with early endosomes containing EEA1 and Rab5. Furthermore, we show that functional interference with Rab5 led to accumulation of Nsg1 and Nsg2 on the cell surface, suggesting that Rab5 was involved in regulating the endocytosis of this class of somatodendritic proteins. Different effects of Rab5-DN expression have been reported for different cargos and in different cell types. Early reports showed that Rab5 regulated endocytosis of a number of receptors in non-neuronal cell, including TfR^30–32^. Additional roles for Rab5 were identified for EGFR transport out of the early endosome to the late endosome^33^, and in regulating early endosomes^19^. In neurons, less is known. A recent report described mislocalization of many somatodendritic receptors to the axon in cells expressing Rab5-DN^21^, suggesting that Rab5 played a critical role in maintaining the polarized distribution of these receptors by retrieval of mislocalized receptors from the axon. We, on the other hand, observed no axonal mislocalization for either Nsg1 or Nsg2 in Rab5 interference experiments. Endocytosis of Nsg1 and Nsg2 thus is regulated similarly to TfR and depends on Rab5 activity.

### Rab7 regulates Nsg1 and Nsg2 transport

Both steady-state and endocytosed Nsg1 and Nsg2 were found to co-localize with Rab7-endosomes and to co-travel with Rab7 by live imaging. This is the first evidence that Nsg1 and Nsg2 localize to late endosomes. Rab7 regulates progression from early endosomes to late endosomes, as well as maturation steps between late endosomes to lysosomes^33–35^. We found that inhibition of Rab7 function led to accumulation of endocytosed Nsg1 and Nsg2 and that endosomal flux of Nsg1 and Nsg2 is thus regulated by Rab7.

Using triple live imaging in combination with endocytosis assays for Nsg1, we found that endocytosed Nsg1 was either stationary or moved bi-directionally in dendrites with only a slight bias towards retrograde trafficking. This is consistent with other work in which strong directional bias was not seen in dendrites for endocytosed Tfn or endocytosed L1/NgCAM^5^. However, at least one cargo, BACE^36^, undergoes preferential retrograde transport in dendrites after endocytosis in Rab11-endosomes. In contrast, Nsg1 and Nsg2 were largely excluded from Rab11-endosomes. Some cargos thus are able to preferentially engage retrograde motors after entering dendritic endosomes. How directionality of cargos is controlled in dendrites is an open question since microtubules are arrayed in mixed polarity in dendrites^37, 38^. Recent work has implicated KIF21B in retrograde dendritic transport of BDNF-TrkB containing endosomes, but these were not Rab7-positive^39^.

### Nsg1/2 rapidly degrade after endocytosis

Half-lives vary greatly between different membrane proteins and can be as short as one hour^40^ and as long as several weeks^41^. The overwhelming majority of synaptic proteins have half-lives of 2–5 days^42^. The cellular and molecular mechanisms that regulate how quickly membrane proteins turn over are under active investigation since they are important contributors to cellular homeostasis^43–45^. This is also true in neurons where disturbance of proteostasis is thought to be at the root of numerous neurodegenerative disorders^46^. We found surprisingly that Nsg1 and Nsg2 have half-lives of under two hours. The rapid flux towards lysosomes suggest that Nsg1 and Nsg2 do not contain targeting motifs to remain resident in dendritic endosomes. The steady state distribution of Nsg1 and Nsg2 thus reflects the kinetics of their endocytosis into early endosomes, trafficking to late endosomes and degradation in lysosomes. This finding is consistent with ultrastructural detection of Nsg1 in multivesicular bodies^2, 47^. The short half-lives of this family was quite surprising because knockdown of Nsg1 causes mistrafficking of multiple receptors which are not themselves short-lived. It remains an important question for the future to determine how the short-lived Nsg1 and Nsg2 proteins regulate trafficking of much longer-lived dendritic receptors.

## Materials and Methods

### Antibodies

Anti-Nsg1 antibody using the same peptide sequences against the N-terminal of Nsg1 from *aa* 7 to 23 (NFAEKGTKQPLLEDGFD) as the one in Steiner *et al*.^4^, was raised in rabbit by Open Biosystem, Thermo Fisher and in goat by Everest Biotech with peptide sequence from aa 7 to 20 (1:300 IF, cat#EB11637, RRID:AB_2554545).

Anti-Nsg1 (goat polyclonal) against the C-terminal region of Nsg1 from aa 148 to aa 161 (NHYNVAKQSITRSV) was raised in goat by Everest Biotech. The specificity of this antibody was validated by detecting overexpressed Nsg1 in HEK293 cells (Supplementary Figure 1). This antibody only works in live cell staining and it recognizes the surface localization of endogenous Nsg1 in neurons and of overexpressed Nsg1 in transfected cells.

Anti-Nsg2 rabbit polyclonal against the cytoplasmic region of Nsg2 from aa 138–155 (SHYSVAKQSTARAIGPWLS) was generated by Open Biosystem, ThermoFisher. We validated the specificity of this antibody by WB (1:1000) using overexpressed cells and brain lysates, where a band at 19KD was detected, and by shRNA knockdown^13^.

Anti-Nsg2 goat polyclonal against the C-terminus tail of Nsg2 from aa 161 to 171 (HEPKPPKTQGH) by Everest Biotech (1:400 IF, cat#EB12967). We validated the specificity of this antibody by WB and knockdown of the protein by shRNA.

Anti-Nsg2, rabbit monoclonal, 1:500 IF, cat#ab18953, Abcam, RRID: AB_2571866; Anti-EEA1, mouse monoclonal, 1:100 IF, 1:1000 WB, cat#610456, BD Biosciences, RRID: AB_399409; anti-GM130, mouse monoclonal, 1:500 IF, cat#610822, BD Biosciences, RRID: AB_1645351; anti-TGN38, mouse monoclonal, 1:200 IF, cat#610899, BD Biosciences, RRID: AB_398216; Anti-Lamp1, mouse monoclonal,1:2000 IF, cat#ADI-VAM-EN001, Enzo Life Sciences, RRID:AB_2038958; Rab5, rabbit monoclonal, 1:200 IF, 1:1000 WB, cat#3547, Cell signaling, RRID: AB_2300649; Rab7, rabbit monoclonal, 1:100 IF, 1:1000 WB, cat#9367, Cell Signaling, RRID:AB_1904103; anti-Rab11, rabbit monoclonal, 1:200 IF, cat#5589, Cell Signaling, RRID:AB_10693925; anti-M6PR (Mannose-6-phosphate receptor), rabbit monoclonal, 1:200 IF, Cell signaling, cat#14364; *Anti-MAP2*, chicken polyclonal, 1:2000 IF, cat#CPCA-MAP2, EnCor Biotechnology, RRID:AB_2138173; Anti-beta III tubulin, chicken polyclonal, 1:7500 WB, cat#TUJ, Aves Lab; Alexa 594 anti-GFP rabbit polyclonal, 1:500 IF, cat#A21312, Molecular probes, RRID:AB_221478.

#### Secondary antibodies

Alexa-dye coupled antibodies (Molecular Probes) were used for Immunofluorescence. Cy3-ChromPure Rat transferrin, cat#012-160-050, RRID: AB_2337149, Jackson ImmunoResearch. For Licor Odyssey Western blots, Jackson ImmunoResearch antibodies were used: Donkey anti mouse (680) #715-625-151, Donkey anti rabbit (790) #711-655-152, Donkey anti-goat (800) (Licor) #926-32214.

### Plasmids

*Nsg1-Emerald*:Addgene cat#54202, Michael Davidson lab; the mutation at residue 114 from D to G was corrected.

*Nsg1-mCherry*:mouse full length *Nsg1* was cloned at the N-terminal of mCherry in pcDNA3 vector.

*Nsg2-GFP*:mouse full length Nsg2 was cloned into pcDNA-GFP by Genscript

*mRFP-Rab5*:Addgene cat#14437, Ari Helenius lab.

*mCherry Rab5CA(Q79L) cat#35138, mCherry Rab5DN(S34N) cat#35139*:*Addgene*, Sergio Grinstein lab.

*EGFP-Rab7 cat#12605, EGFP-Rab7DN(T22N) cat#12660*:*Addgene*, *Richard Pagano lab*

*mTagBFP2-Rab5*, cat#56417, Addgene, Michael Davidson lab;

*mTagBFP2-Rab7*:Michael Davidson lab.

*GFP*:Clontech

*mCherry*:Clontech.

### Neuronal cultures

Neuronal cultures were prepared from E18 rat hippocampi, as approved by the University of Virginia Animal Care and Use Committee. All experiments were performed in accordance with relevant guidelines and regulations (ACUC protocol #3422). Hippocampi from all pups in one litter were combined and thus contained male and female animals. Cells were plated on poly-L-lysine coated coverslips and incubated with plating medium containing DMEM medium with 10% horse serum. For live imaging use, neurons were plated on a 35mm glass bottom microwell dish (MatTek). After 4 h, the plating medium was removed and replaced with serum-free medium supplemented with B27 (ThermoFisher), and neurons were cultured for 7–10 DIV (days *in vitro*) for experimental use. Transfections were carried out with Lipofectamine 2000 (Invitrogen). Neurons were transfected with either mRFP-Rab5, mCherry-Rab5CA, mCherry-Rab5-DN, GFP-Rab7 or GFP-Rab7-DN for 36–40 hours. All transfection experiments were repeated in five to ten independently derived cultures. At last 25 fields were captured per experiment. Experiments using endogenous localization (Tfn, Rab11, Rab7, EEA1, Rab5) were repeated in three independent cultures. All images and line scans are representative examples that are highly reproducible and robust.

### Immunocytochemistry

Cells were fixed in 2% paraformaldehyde/3% sucrose/PBS in 50% conditioned medium at room temperature for 30 minutes, quenched in 10 mM glycine/PBS for 10 minutes. The fixation conditions used do not introduce holes into the overwhelming majority of cells. Coverslips were then blocked in 5% horse serum/1% BSA/PBS ± 0.2% TritonX-100 or 0.1% saponin for 20 minutes. Antibodies were diluted in 1% BSA/PBS and incubated for 1 hour. For all surface staining, live cells were incubated for 30 min at 37 °C in primary antibody before fixation, and secondary antibody was applied before permeabilization. Coverslips were mounted in Prolong Gold mounting medium and viewed on a Zeiss Z1-Observer with a 40x objective (EC Plan-Neofluar 40x/0.9 Pol WD = 0.41). Apotome structured illumination was used for most images. Images were captured with the Axiocam 503 camera using Zen software (Zeiss) and processed identically in Adobe Photoshop. No non-linear image adjustments were performed. Confocal imaging of fixed samples was carried out on an inverted Zeiss LSM880 confocal microscope using 40X oil objective (LD-C Apochromat 1.3 NA). Airyscan images were collected using 63X objective (NA 1.4) on an inverted Zeiss LSM880 confocal microscope with an Airyscan detector. Airyscan imaging enables about 2-fold better spatial resolution in all three dimensions compared to conventional laser scanning confocal microscopy.

### Line scans

Line scans were used to illustrate the extent and precision of co-localization, similarly to other work^6, 17, 18^. Line scans allow the reader to assess multiple and more diverse aspects of the co-staining than can be captured from a single calculated coefficient of co-localization. They allow assessment not only of position, but also of the relative brightness of different markers. This is particularly informative for endosome markers that are dynamically recruited onto endosomes as they mature, such as Rabs and EEA1. Endosomes with bright EEA1 staining, for instance, are at a different maturation stage than endosomes with only faint EEA1 staining^6, 48^. Line scans are thus the best way to represent the variety of compartment distributions we observe.

### Quantification of co-localization

For Nsg1 and Nsg2 co-localization with EEA1, rab11, rab7, and LAMP1, Z-stacks were collected by confocal imaging. Individual fields of view were analyzed using Imaris image analysis software for each antibody counted. All analysis was done on 3D voxels. 1 to 5 dendrites per field of view were selected and masked based on MAP2. Dendrites that were distinguishable and not overlapping with other staining were selected. This is critical because all of the endosomal markers are also expressed in the non-neuronal cells growing in the cultures. Surface objects based on Nsg1 or Nsg2 staining were created in the masked regions. These objects were then used to mask a second region – an “Nsg1 or Nsg2 dendrite ROI”. Thresholds for the secondary markers were then set to subtract non-specific background, and the percent of voxels within the “Nsg1 or Nsg2 dendrite ROI” that were also positive for the endosomal markers was calculated.

For Nsg1/Nsg2 endosomal co-localization, individual fields of view were analyzed from confocal Z-stacks using Imaris image analysis software. The somata were masked and then excluded from the analysis because Nsg1 and Nsg2 are prominent in the TGN. Spot objects for all Nsg1 and Nsg2 positive endosomes were created and the distance to their nearest neighbor was calculated. All objects with a nearest neighbor less than 0.5 µm center to center were counted as co-localized. The number reported is the percent of the total spots that fit the co-localization criterium.

For quantifying intensity changes of endocytosed Nsg2 over time, the ImageJ-based Fiji software was used. Images from independent experiments were treshholded to the same intensity to subtract non-specific background identically for all images. Objects corresponding to endosomes were identified using identical parameters for all images. The number and intensity of identified objects in each field were quantified using Fiji. 24–26 fields were quantified per time point. Each field contained many cells and the total counts represent over 600 cells.

### Endocytosis Assay

Neurons (DIV8-10) were incubated with anti-Nsg1 or anti-Nsg2 antibodies against endogenous proteins for 30 min at 37 °C. For observing retention of endocytosed Nsg1/2 in neurons transfected with Rab5CA or Rab7-DN, cells including those transfected with Rab5 or Rab7 WT or GFP were incubated with anti-Nsg1 and anti-Nsg2 antibodies for 2 hrs. For observing co-endocytosed Nsg2 with transferrin, neurons were first incubated in serum-free medium in the presence of N2 supplement and ovalbumin for 30 minutes, followed by incubation with anti-Nsg2 and rat transferrin-conjugated with Cy3 (20 µg/ml) (Jackson Immunoresearch) for another 30 minutes. To reduce surface–bound antibody without damaging the cells, we washed neurons with PBS twice, followed by a one-minute treatment with pH 4.0, MEM, followed by two more PBS washes before returning to the incubator for various time points. To ensure no remaining surface-bound antibody that might be misinterpreted as internalized antibody, neurons were washed once with pH 2.0 MEM for 30 seconds before fixation in 2% paraformaldehyde/3%sucrose/PBS, pH 7.4. Internalized antibody was detected by applying secondary antibodies after permeabilization.

### Live Imaging

Live imaging was carried out essentially as described^49^ with slight modifications. Briefly, neurons at DIV7 were transfected for 36 to 40 hrs with the following plasmids combinations: *Nsg1-Emerald* with *mRFP-Rab5*, *Nsg1-mcherry* with *GFP-Rab7*, *Nsg1-mcherry* with *Nsg2-GFP*, *Nsg1-Emerald* with *mTagBFP2-Rab5*, *Nsg1-Emerald* with *mTagBFP2-Rab7*. Neurons were maintained in phenol-red free Neurobasal medium. Prolong Live anti-fade (Invitrogen Life technologies) was added right before live imaging. For imaging the endocytosed *Nsg1-Emerald*, transfected neurons were incubated with Alexa-594 conjugated anti-GFP antibody for 30 minutes, washed several times with PBS, and then proceed to live imaging on a heated stage in a chamber with 5% CO_2_. All dual and triple live imaging was conducted on an inverted Zeiss LSM880 confocal microscope using 40X water objective (LD-C Apochromat 1.2 W). Images from dual or triple channels were acquired simultaneously with bidirectional scan-frame mode every second for 300–500 frames and with tight gate settings to reduce any overlapping fluor spectra. Laser lines at 405 nm for BFP, at 488 nm for GFP/Emerald, and at 594 nm for mCherry expression were used. Kymographs were made using FIJI functions, as in ref. 6. Live imaging for any given set of transfected constructs was repeated in at least five independent cultures. Each time, at least four cells were imaged live.

### Western blot

Neurons at DIV10 were treated with cycloheximide (20 µg/ml) for 1, 2 and 4 hrs, washed several times with PBS, and harvested for subcellular fractionation using BioVision FractionPREP kit following the protocol described by the manufacturer. The cytosolic and membrane fractionated samples were subjected to SDS-PAGE and western blot analysis.

### Data Availability

All data generated or analyzed during this study are included in this published article (and its supplementary information files). Data not shown are available from the corresponding authors upon a reasonable request.

## Electronic supplementary material

Supplementary Information
