# Supplementary material for: The endosomal neuronal proteins Nsg1/NEEP21 and Nsg2/P19 are itinerant, not resident proteins of dendritic endosomes
Source: Sci Rep. 2017 Sep 5;7:10481. doi: 10.1038/s41598-017-07667-x (PMC5585371; doi:10.1038/s41598-017-07667-x)
Supplement: Supplementary file 1 — Supplementary Information [file 41598_2017_7667_MOESM1_ESM.pdf]

Supplementary Information for
"The endosomal neuronal proteins Nsg1/NEEP21 and Nsg2/P19 are itinerant, not resident proteins of dendritic endosomes."
Chan Choo Yap, Laura Digilio, Lloyd McMahon, Bettina Winckler

Suppl. Figure S1: *Validation of Ct-directed anti-Nsg1 antibody.*

HEK293 cells were transfected with Nsg1-Em or Nsg2-GFP (as negative control) and stained with anti-Ct-Nsg1 antibody. Both Nsg1-Em and Nsg2-GFP are found in intracellular compartments and on the cell surface when overexpressed in HEK 293 cells. Since this antibody can only detect its epitope in live cells, surface staining of live cells was performed. Only cells expressing Nsg1-Em (green) showed staining with the anti-Nsg1 antibody (red) whereas non-transfected cells (visualized with DAPI in blue) or cells expressing Nsg2-GFP were not stained.

Fig S1

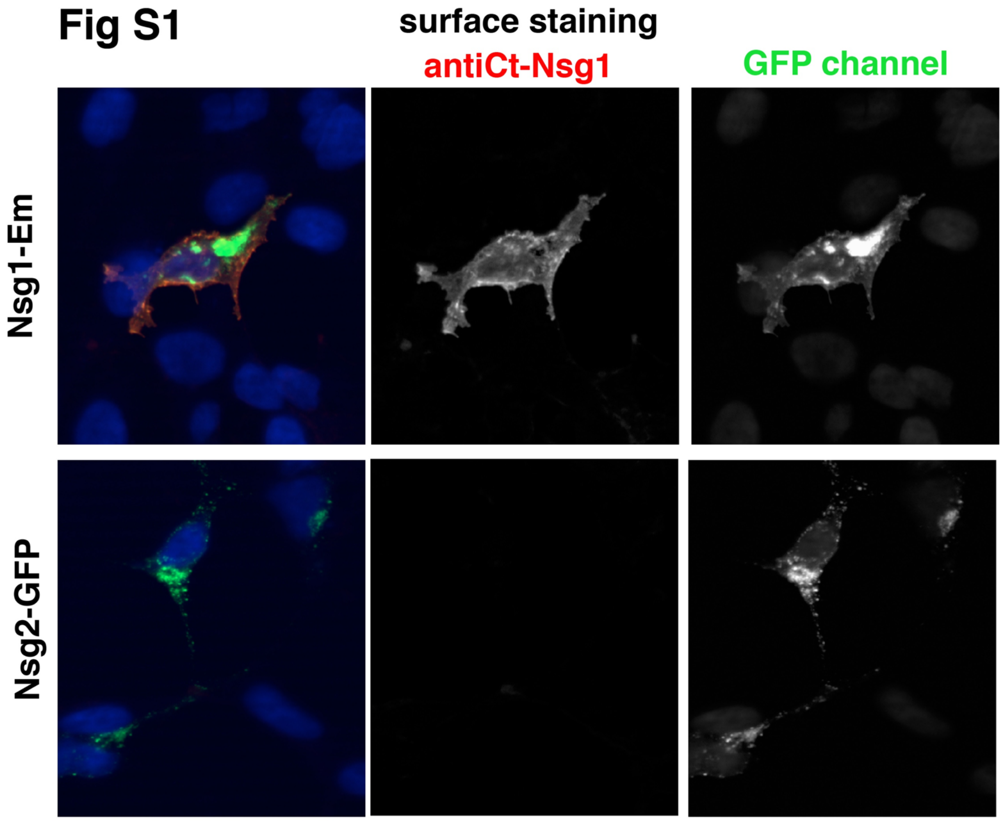

<u>Suppl. Figure S2:</u> *Nsg2 co-localizes with TGN38 in the soma* (related to Figure 1). Cultured neurons were stained against endogenous Nsg2 and TGN38. There is extensive co-localization in the soma.

Fig. S2

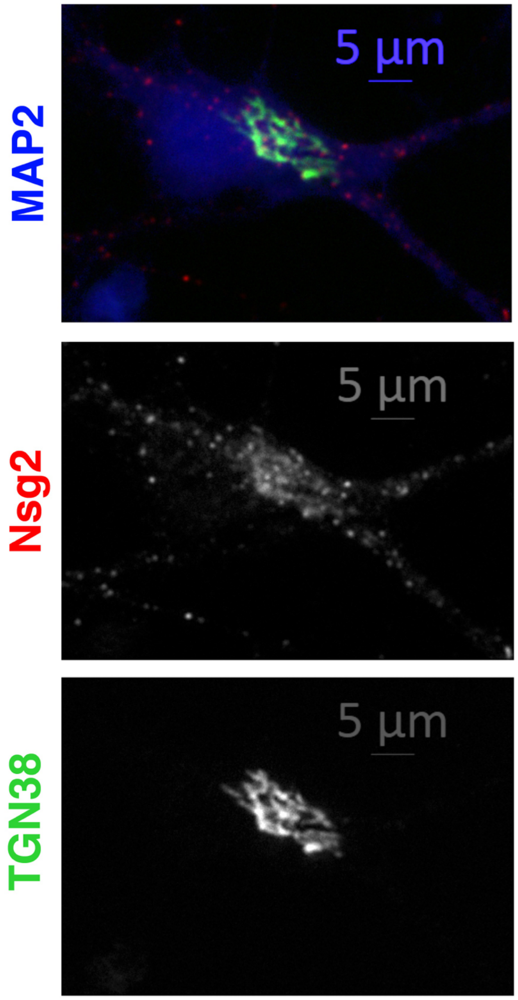

Suppl. Figure S3: *Faint surface staining of endogenous Nsg1 and Nsg2 can be detected in cultured hippocampal neurons* (related to Figure 1).

Live neuronal cultures were incubated with anti-Ct directed antibodies against endogenous Ngs1 and Nsg2 to detect surface pools, and then washed and fixed prior to permeabilization for immunostaining with secondary antibody and counterstained against endogenous MAP2 and EEA1.

Fig. S3

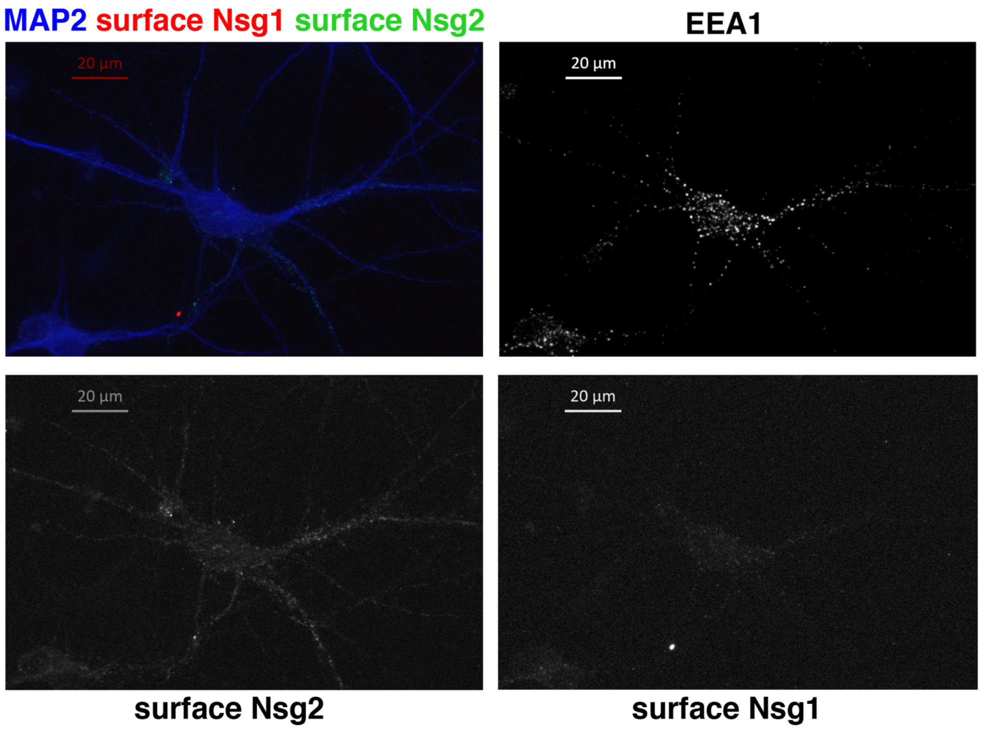

<u>Suppl. Figure S4:</u> *Total pool of endogenous Nsg1 and Nsg2 appears less punctate in neurons expressing Rab5-DN* (related to Figure 3C).

Cultured neurons were transfected with Rab5-DN-GFP (green) and cultures fixed and stained against MAP2 and endogenous Nsg1 and Nsg2 after permeabilization to reveal the total pools of Nsg1 and Nsg2. Arrow designates the neuron transfected with Rab5-DN-GFP. Arrowheads designate untransfected neurons in the same field. The TGN-like somatic staining and the punctate endosomal staining prominent in the non-transfected cells are less apparent with more diffuse appearance of the staining in the transfected cell. Single channels as well as merged images are shown in the panels, as marked underneath.

Fig. S4

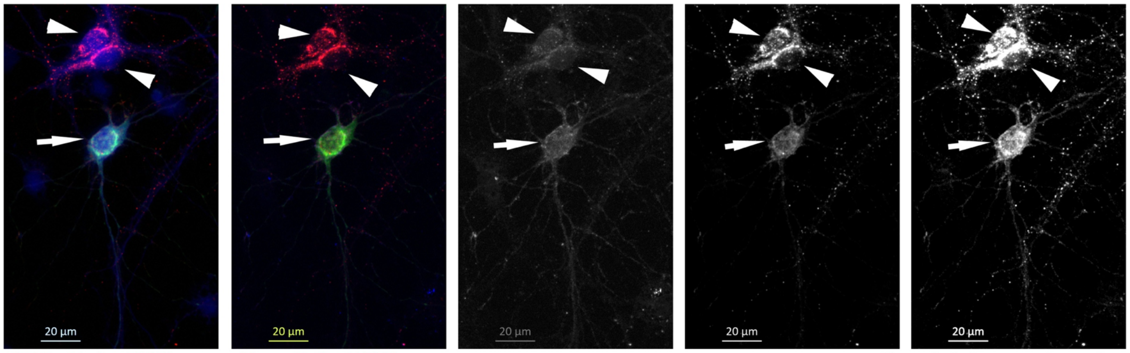

MAP2 Rab5-DN Nsg2 | Nsg1 Rab5-DN Nsg2 | Nsg1 | Nsg2 | Nsg2 brighter to show dendrites

Suppl. Figure S5: *Effects of expressing Rab5-CA (constitutively active) or Rab5-DN (dominant negative) on the distribution of Golgi and endosome proteins* (related to Figure 3D).
Neurons expressing Rab5-DN or Rab5-CA (red) were counterstained with Nsg1 (green) and either the Golgi marker GM130 (A; blue), the TGN-endosome protein M6PR (B; blue), or the lysosome protein LAMP1 (C; blue). In each panel, the transfected cell is marked with an arrow. Non-transfected cells are marked with arrowheads for comparison in the same field. Small arrows in C show co-localization of LAMP1 with Rab5-CA. Size bar = 10 μm.

Fig. S5

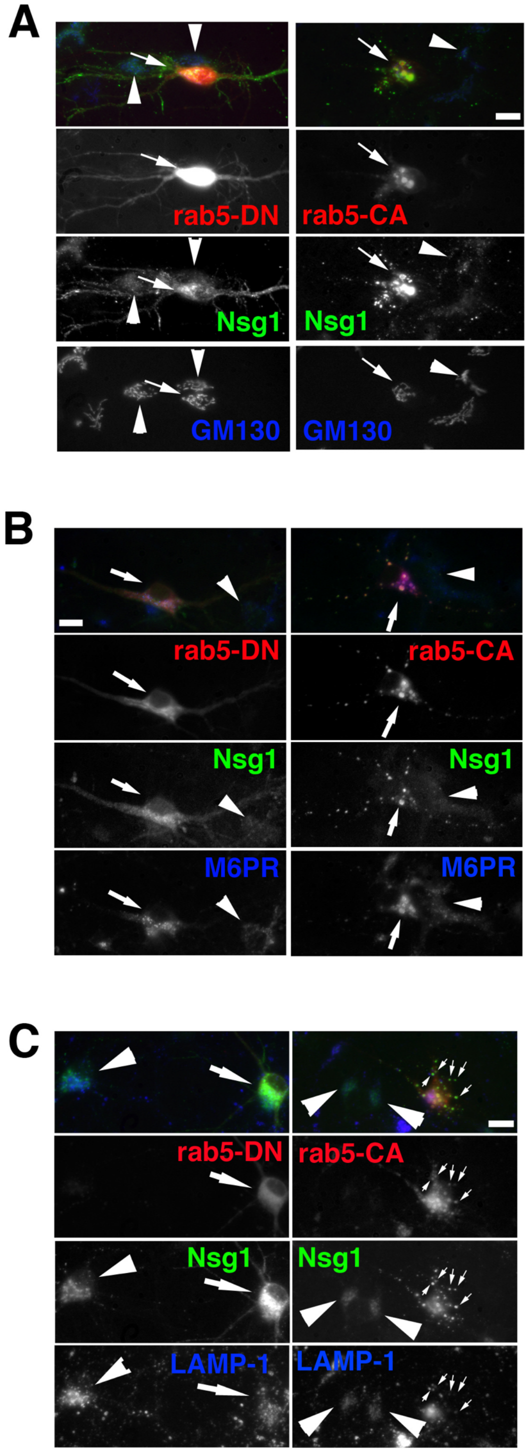

Suppl. Figure S6: *Endocytosed Nsg1 localizes to EEA1 early endosomes and occasionally to EEA1 endosomes containing Rab11* (related to Figure 4A-C).

Neurons were incubated with anti-Nsg1 antibody live and then washed. Cells were either fixed immediately (t=0) (A) or after 25 minutes of chase (t=25 min) (B). Staining was carried out as indicated on the panels. The area below the white line is shown as insets with channels separated singly or in combinations of two markers, as indicated. Red arrows point at Rab11 endosomes. Blue arrows point at EEA1 endosomes. The position of EEA1 and Rab11 endosomes (as marked by arrows) is overlaid over the endocytosed Nsg1 channel for comparison of localization. White arrows indicate compartments containing all three markers. Note that when endocytosed Nsg1 was found co-localized with Rab11, EEA1 was also present, indicating that this compartment was still a maturing early endosome.

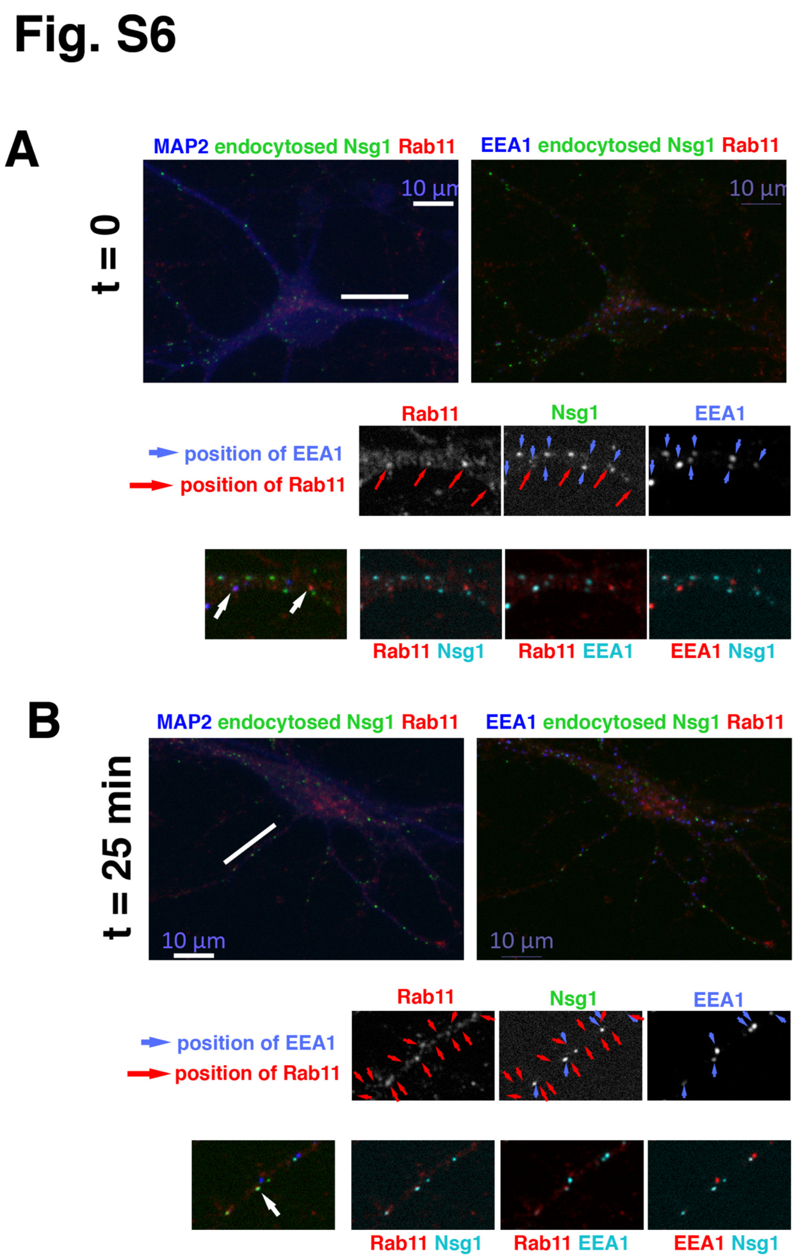

Suppl. Figure S7 A: *Knockdown of Rab7 with shRab7 plasmid causes brighter accumulation of Nsg1 and Nsg2 in endosomes, similarly to Rab7-DN* (related to Figure 5F).

Neuronal cultures were transfected for 5 days with shControl-GFP or shRab7-GFP and stained for endogenous Nsg1 (red) and Nsg2 (blue). The transfected cell is marked with a green arrow. Singe channel images are also shown. MAP2 counterstain is shown in the bottom panels to visualize the position of untransfected neurons. Both Nsg1 and Nsg2 accumulate to much higher levels in the shRab7-GFP expressing neuron compared to either untransfected cells or cells transfected with shControl-GFP.

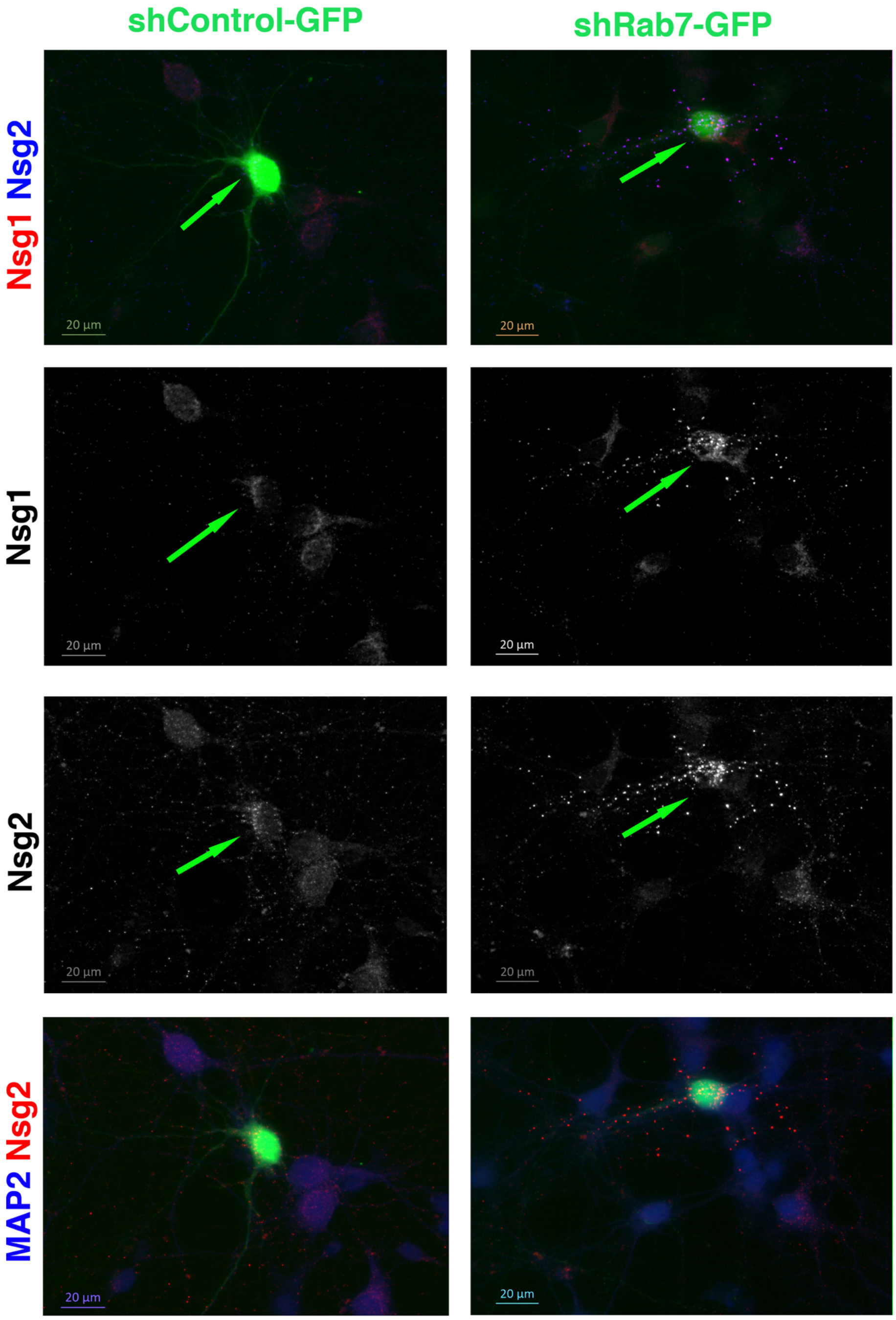

Fig. S7A

<u>Suppl. Figure S7 B</u>: *Expression of Rab7-DN causes brighter accumulation of Nsg1 and Nsg2 in endosomes* (related to Figure 5F).

Neuronal cultures were transfected for 36-40 hours with WT GFP-Rab7 (left panels) or GFP-Rab7-DN (right panels) and stained for endogenous Nsg1 (red) and Nsg2 (blue). The transfected cell is marked with a green arrow. Singe channel images are also shown. MAP2 counterstain is shown in the bottom panels to visualize the position of untransfected neurons. Both Nsg1 and Nsg2 accumulate to much higher levels in the Rab7-DN expressing neuron compared to either untransfected cells or cells transfected with WT GFP-Rab7.

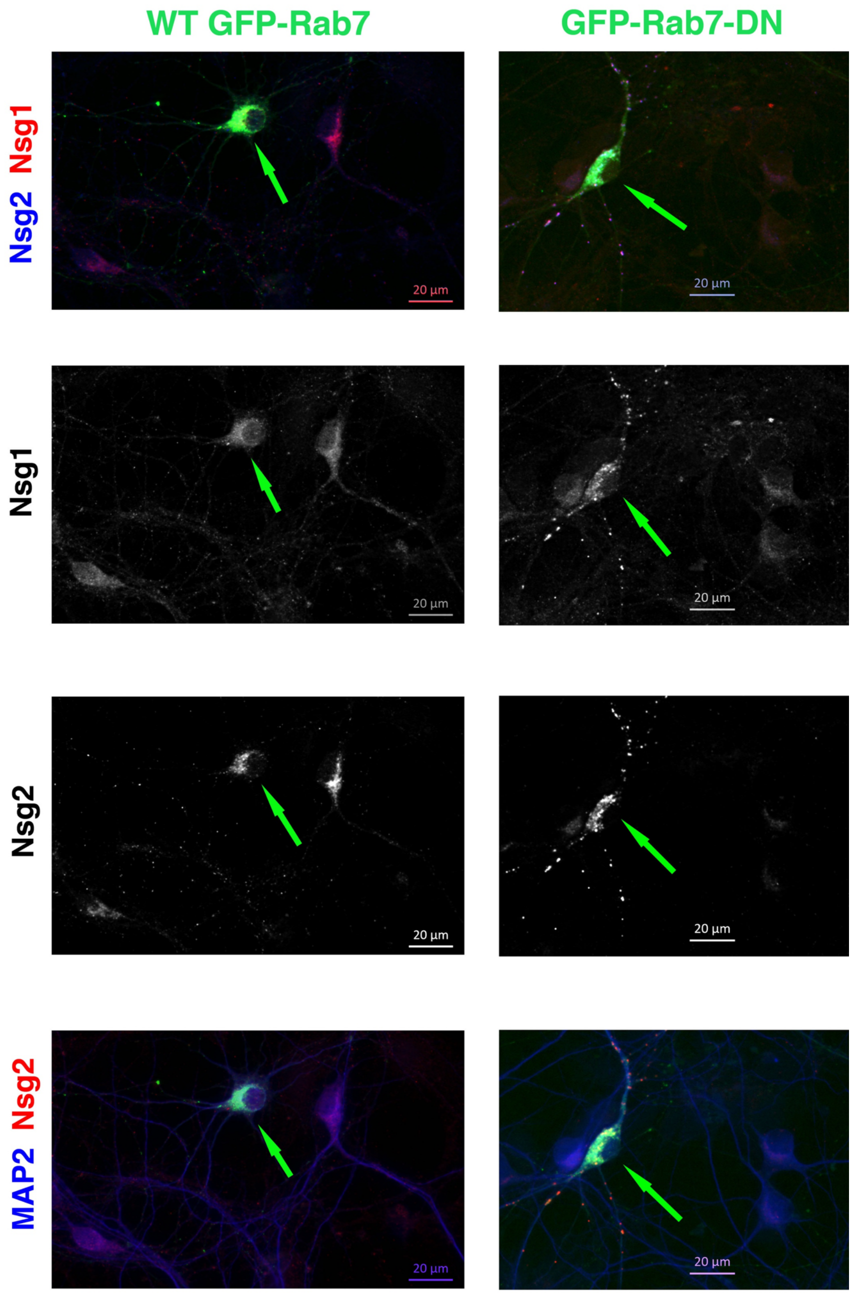

Fig. S7 B

<u>Suppl. Figure S8:</u> *Full length blots for the cropped lanes shown in Figure 6F*. Hippocampal cultures were treated with cycloheximide for 0-4 hours as indicated below the lanes and then lysates were fractionated. The membrane/cytoskeletal fractions were separated on SDS-PAGE and probed with antibodies against β3-tubulin, Rab7, EEA1, Nsg1 and Nsg2, as labelled. Molecular weight markers are indicated on the left.

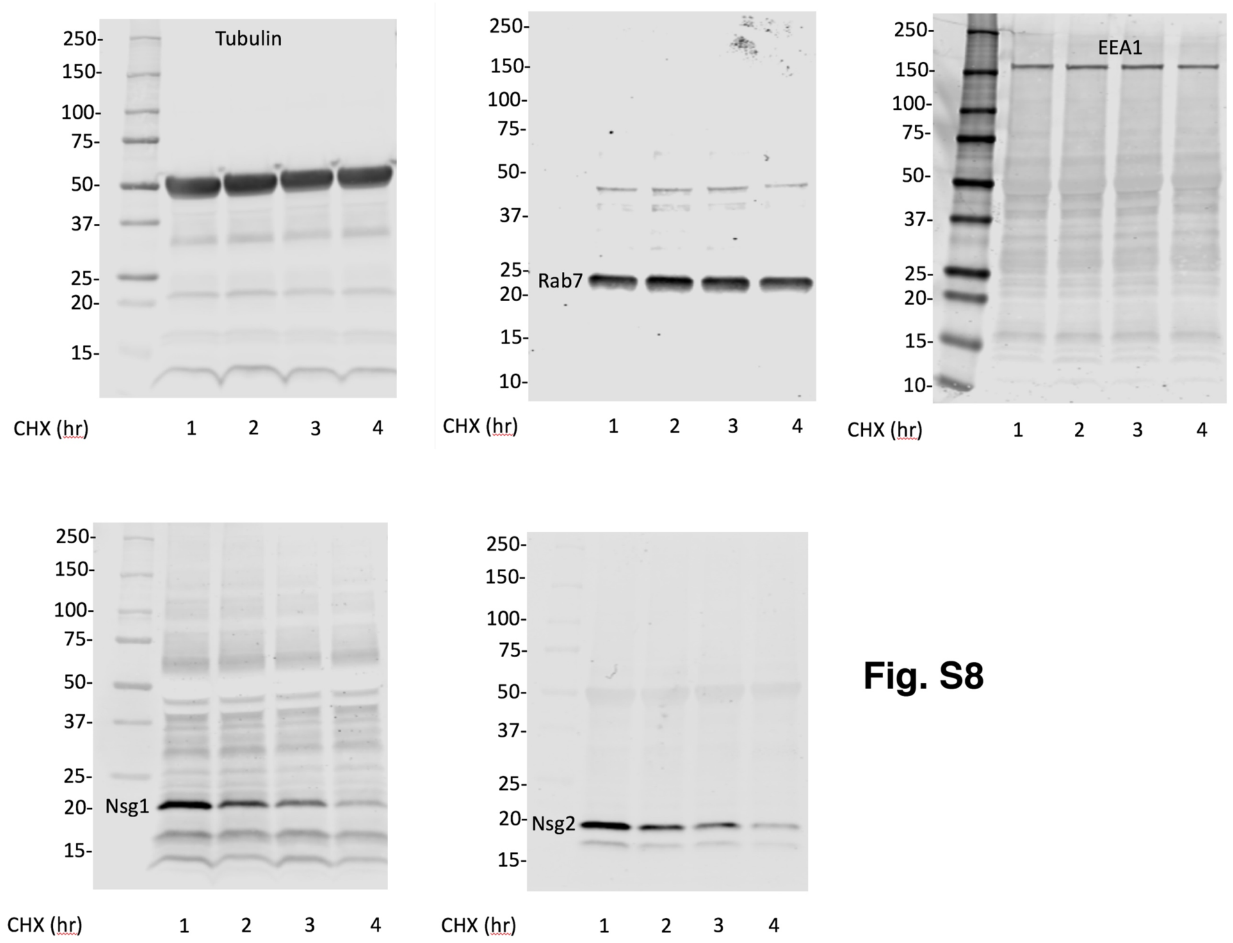

Fig. S8
